# TEX264 coordinates p97- and SPRTN-mediated resolution of topoisomerase 1-DNA adducts

**DOI:** 10.1038/s41467-020-15000-w

**Published:** 2020-03-09

**Authors:** John Fielden, Katherine Wiseman, Ignacio Torrecilla, Shudong Li, Samuel Hume, Shih-Chieh Chiang, Annamaria Ruggiano, Abhay Narayan Singh, Raimundo Freire, Sylvana Hassanieh, Enric Domingo, Iolanda Vendrell, Roman Fischer, Benedikt M. Kessler, Timothy S. Maughan, Sherif F. El-Khamisy, Kristijan Ramadan

**Affiliations:** 1 Cancer Research UK and Medical Research Council Oxford Institute for Radiation Oncology, Department of Oncology, University of Oxford, Oxford, OX3 7DQ UK; 2 The University of Sheffield Neuroscience Institute and the Healthy Lifespan Institute, Department of Molecular Biology and Biotechnology, Firth Court, University of Sheffield, S10 2TN Sheffield, UK; 3 Unidad de Investigación, Hospital Universitario de Canarias, Ofra s/n, La Cuesta, 38320 La Laguna, Tenerife Spain; 4 Instituto de Tecnologías Biomédicas, Universidad de La Laguna, 38200 La Laguna, Tenerife Spain; 5 Universidad Fernando Pessoa Canarias, 35450 Las Palmas de Gran Canaria, Spain; 6 TDI Mass Spectrometry Laboratory, Target Discovery Institute, Nuffield Department of Medicine, University of Oxford, Oxford, UK

**Keywords:** DNA, Proteolysis

## Abstract

Eukaryotic topoisomerase 1 (TOP1) regulates DNA topology to ensure efficient DNA replication and transcription. TOP1 is also a major driver of endogenous genome instability, particularly when its catalytic intermediate—a covalent TOP1-DNA adduct known as a TOP1 cleavage complex (TOP1cc)—is stabilised. TOP1ccs are highly cytotoxic and a failure to resolve them underlies the pathology of neurological disorders but is also exploited in cancer therapy where TOP1ccs are the target of widely used frontline anti-cancer drugs. A critical enzyme for TOP1cc resolution is the tyrosyl-DNA phosphodiesterase (TDP1), which hydrolyses the bond that links a tyrosine in the active site of TOP1 to a 3’ phosphate group on a single-stranded (ss)DNA break. However, TDP1 can only process small peptide fragments from ssDNA ends, raising the question of how the ~90 kDa TOP1 protein is processed upstream of TDP1. Here we find that TEX264 fulfils this role by forming a complex with the p97 ATPase and the SPRTN metalloprotease. We show that TEX264 recognises both unmodified and SUMO1-modifed TOP1 and initiates TOP1cc repair by recruiting p97 and SPRTN. TEX264 localises to the nuclear periphery, associates with DNA replication forks, and counteracts TOP1ccs during DNA replication. Altogether, our study elucidates the existence of a specialised repair complex required for upstream proteolysis of TOP1ccs and their subsequent resolution.

## Abstract (web summary)

Eukaryotic topoisomerase 1 (TOP1) regulates DNA topology to ensure efficient DNA replication and transcription. Here, the authors reveal insights into the molecular resolution of topoisomerase 1-DNA adducts by TEX264, p97 and SPRTN.

## Introduction

Topoisomerase 1 (TOP1) resolves DNA topological stress accumulated during DNA replication and transcription. As part of its catalytic cycle, TOP1 forms an intermediate that is covalently bound to DNA, known as a TOP1 cleavage complex (TOP1cc). TOP1ccs are usually transient but can become trapped if TOP1 cleaves near a DNA alteration or is exposed to TOP1 poisons. Due to their bulky structure, TOP1ccs hinder the progression of DNA replication and transcription, and are therefore highly cytotoxic^1–3^.

Endogenous TOP1ccs pose a constant threat to genome stability, as illustrated by the numerous neurodegenerative diseases associated with defective TOP1cc repair^2–8^. The cytotoxicity of TOP1ccs is also exploited in cancer therapy by the widely used class of anti-cancer drugs, known as camptothecins (CPT), which stabilise TOP1ccs by binding the TOP1–DNA interface. CPT derivatives are routinely used to treat ovarian, colon, and lung cancers but resistance is common, underscoring the need to identify molecular biomarkers and determinants of resistance to improve patient stratification and outcomes^9^.

A key enzyme in TOP1cc repair is TDP1, a phosphodiesterase that directly hydrolyses the phosphotyrosyl bond that covalently links TOP1 to the 3′ end of an ssDNA break^10^. This step is necessary to allow re-ligation of the broken DNA strand and ensure genome stability. Mutations in *TDP1* give rise to the neurodegenerative disease, SCAN1^2,4,5^. However, TDP1 alone cannot resolve TOP1ccs since its substrate bond is protected within the bulky TOP1cc structure and is therefore inaccessible to the TDP1 active site^10^. TDP1 is unable to resolve full-length, recombinant TOP1ccs in vitro, however, its activity is enabled if the TOP1ccs are heat-denatured or proteolytically digested^11–15^. This raises the question of how TOP1cc processing upstream of TDP1 occurs in vivo. A deeper understanding of this process could unveil mechanisms of clinical resistance to TOP1 poisons and potential targets of therapeutic intervention.

The proteasome and the metalloproteases SPRTN (in metazoans) and Wss1 (in yeast) are thought to digest the protein component of TOP1ccs^16–19^. In humans, mutations in *SPRTN* that impair its proteolytic activity cause Ruijs–Aalfs syndrome (RJALS), which is characterised by hepatocellular carcinoma and premature ageing^20^. SPRTN-deficient human cells accumulate endogenous TOP1ccs and are sensitive to TOP1cc-inducing agents^19^. *SPRTN* hypomorphic mice accumulate TOP1ccs from an early age, particularly in the liver, and ultimately develop liver tumours^18^. This suggests that SPRTN plays a critical role in processing TOP1ccs.

Both the proteasome and SPRTN are highly pleiotropic and preferentially cleave pre-unfolded substrates and/or unstructured protein regions^19,21,22^. While the proteasome degrades ubiquitinated proteins, it is unclear how SPRTN recognises and processes its substrates, which vary substantially in size and structure^16,19,23^. Altogether, this implies that other factors must exist to confer specificity to, and pre-process, TOP1ccs to enable their proteolytic digestion.

Both Wss1 and SPRTN have motifs that enable them to bind the ATPase p97 (also called VCP in mammals and Cdc48 in yeast)^16,17,24^. Cdc48 is known to counteract TOP1cc accumulation, however, the mechanistic basis for how it achieves this is not well defined^16,17,25^. Here, we demonstrate that p97 is a key player in TOP1cc repair in human cells. We identify the gyrase inhibitory-like protein, TEX264, as a p97 cofactor that recruits p97 to TOP1ccs. TEX264 recognises both unmodified and SUMO1-modified TOP1 and promotes p97- and SPRTN-dependent TOP1cc repair. TEX264 localises to the nuclear periphery, associates with DNA replication forks, and promotes TOP1cc repair during DNA replication. Cells deficient in TEX264 accumulate endogenous TOP1ccs, are sensitive to clinically relevant doses of TOP1 poisons, and exhibit DNA replication stress. Our data suggest that p97 and TEX264 enable the repair of TOP1ccs by facilitating their proteolysis via SPRTN upstream of TDP1. This discovery is important for preserving genome stability from endogenous TOP1ccs and could be relevant for clinical resistance to TOP1 poisons.

## Results

### The ATPase p97 promotes TOP1cc repair

As TOP1ccs are common endogenous DNA lesions, we reasoned that factors that promote their repair should interact with the TOP1 protein even in the absence of TOP1 poisons^1,26^. To identify modulators of TOP1cc repair, we isolated chromatin from YFP-TOP1-expressing human embryonic kidney (HEK) 293 cells and subjected YFP immunoprecipitates to liquid chromatography–tandem mass spectrometry (LC–MS/MS; Source Data). This analysis identified the ATPase p97 as an abundant interacting partner of TOP1 on chromatin, which we confirmed by immunoblotting (Supplementary Fig. 1A). By using energy generated from ATP hydrolysis, p97 remodels its substrates and extracts them from macromolecular structures such as chromatin^27–33^. Given this known role of p97, and since Cdc48 has been implicated in TOP1cc repair, we investigated whether p97 contributes to TOP1cc processing in human cells.

**Fig. 1:**
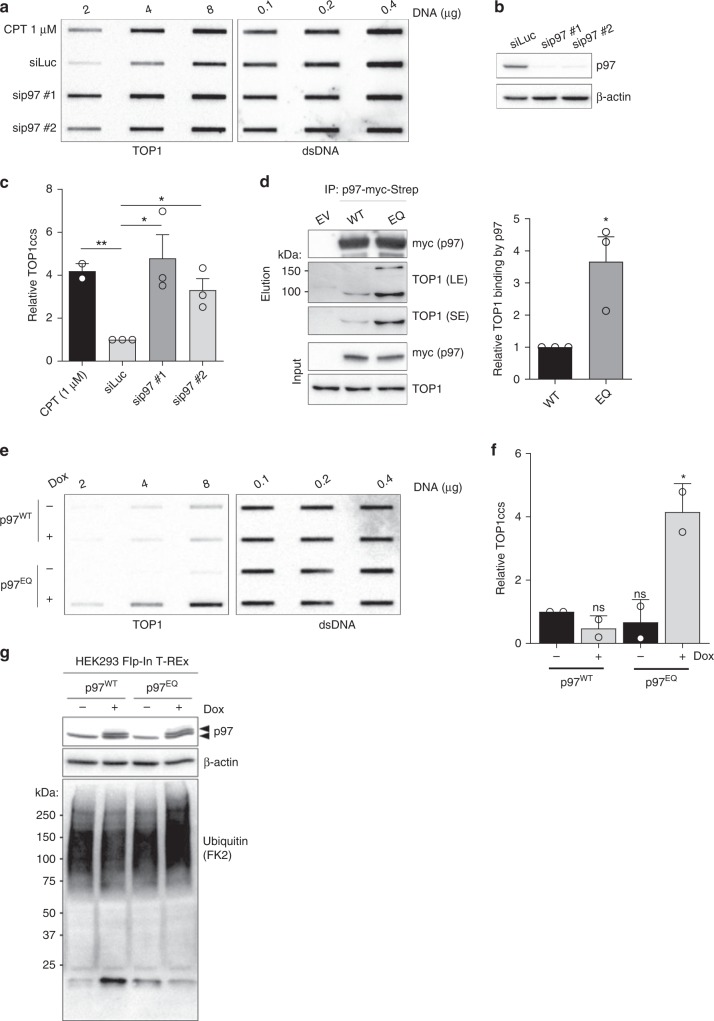
The p97 ATPase promotes TOP1cc repair. **a** RADAR assay to assess TOP1cc accumulation after short interfering (si)RNA-mediated depletion of p97. Treatment with 1 μM CPT for 1 hour was used as a positive control for TOP1cc induction. Double-stranded (ds)DNA is used as a loading control. **b** Immunoblot to confirm p97 depletion. **c** Quantification of A (error bars represent mean ± SEM; *n* = 2 for CPT (1 μM); *n* = 3 for siLuc, sip97 #1 and #2; *P < 0.05; ns, not significant; Student’s *t*-test). **d** Left: immunoblots of anti-Strep-tag immunoprecipitates prepared from HEK293 transiently expressing wild-type (WT) or ATPase-defective (E578Q/EQ) p97-myc-Strep. EV denotes empty vector. LE and SE denote long and short exposure, respectively. Right: quantification of three independent experiments (error bars represent mean ± SEM; *P *<* 0.05; Student’s *t*-test). **e** RADAR assay to assess TOP1ccs in doxycycline (Dox)-inducible HEK293 Flp-In TRex cells expressing the indicated p97 variants. Where indicated, cells were treated with Dox for 36 h. **f** Quantification of **e** (error bars represent mean ± SD; *n* = 2; *P < 0.05; Student’s *t*-test). **g** Immunoblots of whole cell extracts prepared from the same cells as those subjected to RADAR in **e**. Arrowheads indicate endogenous p97 (lower band) and induced p97-myc-Strep (upper band). Source data are available online.

To assess if p97 counteracts TOP1cc accumulation in unchallenged conditions, we employed a modified version of the recently described rapid approach to DNA adduct recovery (RADAR) assay to analyse the abundance of proteins covalently attached to DNA^34^. By lysing cells in chaotropic salts (6 M guanidium isothiocyanate), detergents (4% Triton X-100 and 1% N-lauroylsarcosine), and a reducing agent (1% DTT), all molecular interactions, other than covalent interactions, are disrupted. Depletion of p97 in HEK293 cells with either of two different siRNA sequences resulted in substantial TOP1cc accumulation, to a similar extent as a short treatment with 1 µM CPT (Fig. 1a–c). In co-immunoprecipitation experiments, we found that TOP1 bound ~3-fold more strongly to a substrate-trapping, ATPase-defective p97 variant (E578Q; EQ) than to wild-type p97 (Fig. 1d). This suggested that the TOP1 protein is subject to p97’s ATPase activity.

To test if p97 ATPase activity contributes to TOP1cc repair, we treated human retinal pigmented epithelial (RPE-1) cells with CB-5083, a potent, selective inhibitor of p97 ATPase activity, and visualised TOP1cc foci formation by immunofluorescence using an antibody which specifically recognises TOP1ccs (Supplementary Fig. 1B)^35,36^. We observed that acute chemical inhibition of p97 caused TOP1cc accumulation (Supplementary Fig. 1C, D). To strengthen our conclusion that TOP1cc repair requires p97 ATPase activity, we monitored TOP1cc formation by RADAR in doxycycline-inducible stable cells expressing either wild-type p97 or the dominant-negative EQ variant (+Dox). We observed that TOP1ccs only accumulated in those cells expressing p97^EQ^ (Fig. 1e, f). Short-term induction of p97^EQ^ did not cause a substantial accumulation of ubiquitinated proteins in whole cell extracts, indicating that impaired TOP1cc clearance in these cells was not a secondary consequence of the free ubiquitin pool being depleted (Fig. 1g). These experiments were performed in the absence of CPT treatment, demonstrating that endogenous TOP1ccs are common and can be counteracted by p97. Overall, we conclude that p97 ATPase activity is needed to counteract TOP1cc accumulation in human cells, as is the case for yeast Cdc48^16,17,25^.

### TEX264 recruits p97 to TOP1ccs

**Fig. 2:**
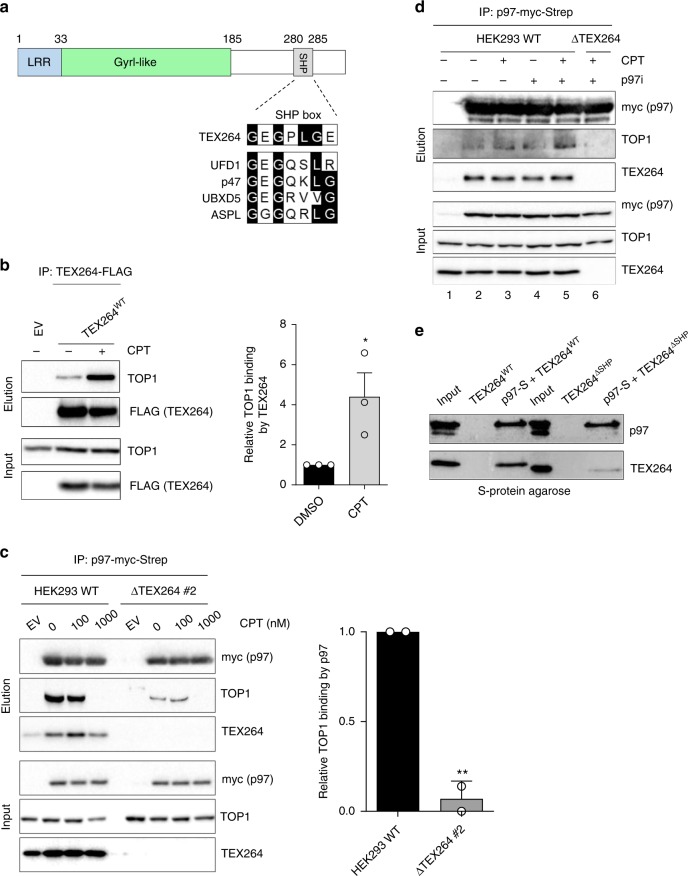
TEX264 bridges p97 to TOP1. **a** Above: schematic diagram of the TEX264 protein. LRR denotes leucine-rich repeat; GyrI-like, Gyrase inhibitory-like domain; SHP, SHP box. Below: sequence alignment of TEX264^280–286^ with the SHP box of other p97 cofactors. Conserved residues are highlighted in black. **b** FLAG immunoprecipitates prepared from HEK293 cells transiently expressing TEX264^WT^-FLAG cDNA or EV, treated with CPT (25 nM) or DMSO for 1 h. Right: quantification of three independent experiments (error bars represent mean ± SEM; **P < 0.01; Student’s *t*-test). **c** Immunoblots of anti-Strep-tag immunoprecipitates prepared from wild-type (WT) or CRISPR-Cas9 *TEX264* knockout (ΔTEX264) HEK293 cells expressing p97-Strep-Myc. Right: quantification of two independent experiments (error bars represent mean ± SD; **P < 0.01; Student’s *t*-test). **d** Immunoblots of anti-Strep-tag immunoprecipitates prepared from wild-type (WT) or ΔTEX264 HEK293 cells expressing p97-Strep-Myc. Cells were treated with DMSO, the p97 inhibitor (p97i), CB-5083 (10 µM), for 90 min, CPT (50 nM) for 60 min, or both, as indicated. **e** In vitro p97 pulldown experiments after incubation of recombinant p97-S tag with His-tagged TEX264^WT^ or TEX264^ΔSHP^. Source data are available online.

To recognise and process its diverse substrates, p97 associates with cofactors which directly bind to p97 via conserved p97 interaction motifs, and typically bridge p97 to ubiquitinated substrates through ubiquitin-binding domains^37,38^. To identify the p97 cofactor that targets p97 specifically to TOP1ccs, we consulted an ongoing mass spectrometry screen of proteins that interact with p97 inside the nucleus (our unpublished data). A protein which stood out as a potential candidate was Testes-expressed 264 (TEX264; Q9Y6I9) because it possesses a gyrase inhibitory-like (GyrI-like) domain (Fig. 2a). In *E. coli*, GyrI-like proteins inhibit the decatenation activity of the type II topoisomerase, DNA gyrase^39,40^. On closer analysis of the TEX264 protein sequence we identified a putative p97 interaction motif, known as a SHP box, located in its unstructured C-terminus, suggesting that TEX264 could be a p97 cofactor (Fig. 2a). TEX264 also possesses an N-terminal leucine-rich repeat (LRR) that was shown to tether the protein to the membrane of the endoplasmic reticulum (ER)^41^.

To validate a physical association between p97, TEX264, and TOP1 in vivo, p97 was isolated from doxycycline-inducible stable HEK293 cells expressing either p97^WT^- or p97^EQ^-myc-Strep at near endogenous levels (Supplementary Fig. 2A). Endogenous TEX264 and TOP1 were present in anti-p97-myc-Strep immunoprecipitates (Supplementary Fig. 2A). Reciprocal co-immunoprecipitation of TEX264-FLAG confirmed that endogenous p97 and TOP1 form a complex with TEX264 in cells (Supplementary Fig. 2C). The interaction between TEX264 and TOP1 increased markedly in response to treatment with a clinically relevant dose of CPT, indicating that TEX264 is recruited to TOP1ccs, along with p97 (Fig. 2b, Supplementary Fig. 2C).

Two recent studies reported a role for TEX264 in the ER^41,42^. However, in addition to TOP1 and p97, numerous nuclear proteins were found to interact with TEX264 by mass spectrometry^41^. We therefore performed biochemical fractionations to assess the subcellular localisation of TEX264. We found that TEX264 is present in the cytosol, nucleus, and on chromatin (Supplementary Fig. 2B). Endogenous TOP1, which was almost exclusively found in the nuclear/chromatin fractions, readily co-immunoprecipitated with TEX264-FLAG (Supplementary Fig. 2B, C). These results show that a subpopulation of TEX264 is nuclear.

Significantly less TOP1 co-immunoprecipitated with p97-myc-Strep in CRISPR-Cas9 *TEX264* knockout cells (ΔTEX264) than in wild-type cells, demonstrating that TEX264 is required to bridge p97 to TOP1 in vivo (Fig. 2c). We noted that the interaction between p97 and TOP1 was not notably stimulated by CPT treatment, possibly due to the highly dynamic way in which p97 processes its substrates. The p97–TOP1 interaction was enhanced upon treatment with CPT when p97’s ATPase activity was chemically inhibited, and this interaction was also dependent on TEX264 (Fig. 2d). We therefore conclude that p97 is recruited to TOP1ccs by TEX264. All of these interactions are resistant to benzonase and ethidium bromide, indicating that they are not indirectly mediated by nucleic acid.

Importantly, TOP1 did not co-immunoprecipitate with either p97 or TEX264 after treatment with 1 µM CPT for 1 h (Fig. 2c, Supplementary Fig. 2C, lane 4). This dose causes DNA replication fork collapse and double strand break (DSB) formation and far exceeds clinically relevant doses (which are in the nanomolar range)^43–45^. This suggests that TEX264 and p97 may only interact with TOP1 when DNA replication complexes are intact. This is in line with the published literature as, after DSB formation, TOP1ccs are resolved by classical DSB repair pathways and their associated nucleases^46–48^.

We next attempted to reconstitute the p97–TEX264–TOP1 complex in vitro. We first purified human p97 and wild-type TEX264 (TEX264^WT^) from *E. coli*. TEX264 was purified without its hydrophobic N-terminus (i.e. LRR: amino acids 1-33) to improve its solubility. TEX264^WT^ readily bound p97 in vitro (Fig. 2e). TEX264^WT^ could also efficiently associate with recombinant TOP1, whereas direct binding between p97 and TOP1 was weaker (Supplementary Fig. 2D–F). When p97 was pre-incubated with TEX264 prior to the addition of TOP1, we observed a significant increase in the amount of TOP1 in p97 pulldowns (Supplementary Fig. 2F, G). This demonstrates that TEX264 can simultaneously bind both p97 and TOP1 and, thus, physically bridge p97 to TOP1.

TEX264 associates with the N-terminus of p97 (amino acids 1–199), indicating that the interaction is independent of the nucleotide-bound status of p97, as is the case for many p97 cofactors (Supplementary Fig. 2H)^38^. Compared with TEX264^WT^, a TEX264 variant lacking its putative SHP box (TEX264^ΔSHP^; amino acids 273–285), bound much less efficiently to p97 in vitro, suggesting that TEX264 is indeed a p97 cofactor (Fig. 2e).

### TEX264 promotes TOP1cc repair and is epistatic with p97 and TDP1

**Fig. 3:**
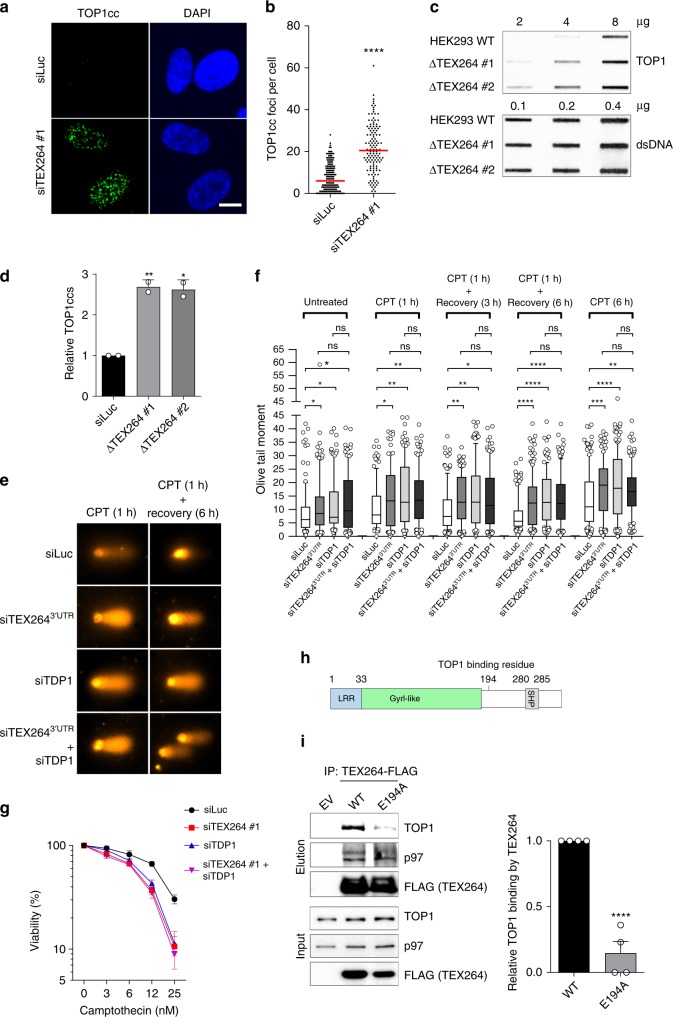
TEX264 counteracts TOP1ccs. **a** Immunofluorescent detection of TOP1ccs (green) in RPE-1 cells transfected with the indicated siRNAs. DAPI 4′,6-diamidino-2-phenylindole. Scale bar, 10 µm. **b** Quantification of **a**. Red line indicates median. Data are representative of two independent experiments. Significance determined by Mann–Whitney test. **c** Slot blot analysis of TOP1ccs prepared from WT or ΔTEX264 HEK293 cells using the RADAR assay. **d** Quantification of **c** (error bars represent mean ± SD; *n* = 2; **P < 0.01, *P < 0.05; Student’s *t*-test). **e** Representative images of cells analysed by alkaline comet assay in **f**. **f** Alkaline comet assay performed in RPE-1 cells treated with the indicated combinations of siRNAs. Cells were treated with CPT (100 nM) for 1 or 6 h(s) and allowed to recover as indicated. Quantification of >100 cells per condition from one representative experiment is shown, and the experiment was repeated two times. Whisker box plots show median values and data within the 10–90 percentile. Statistical analysis was performed using Kruskal–Wallis ANOVA with multiple comparisons, with Benjamini–Hochberg post-test. **g** Colony forming assay to assess the viability of HeLa cells transfected with the indicated siRNAs. Cells were treated for 24 h with the indicated doses of CPT, released into normal media for 7 days, then fixed and stained. Viability represents the number of colonies in each sample expressed as a percentage of the number of colonies formed in the corresponding untreated sample (error bars represent mean ± SD; *n* = 2). **h** Schematic diagram of the TEX264 protein indicating the location of residue E194. **i** Left: Immunoblots of FLAG immunoprecipitates prepared from ΔTEX264 HEK293 cells transiently expressing the indicated versions of FLAG-tagged TEX264. Right: Corresponding quantifications of four independent experiments (mean ± SEM; ****P < 0.0001; ***P < 0.0005; Student’s *t*-test). Source data are available online.

**Fig. 4:**
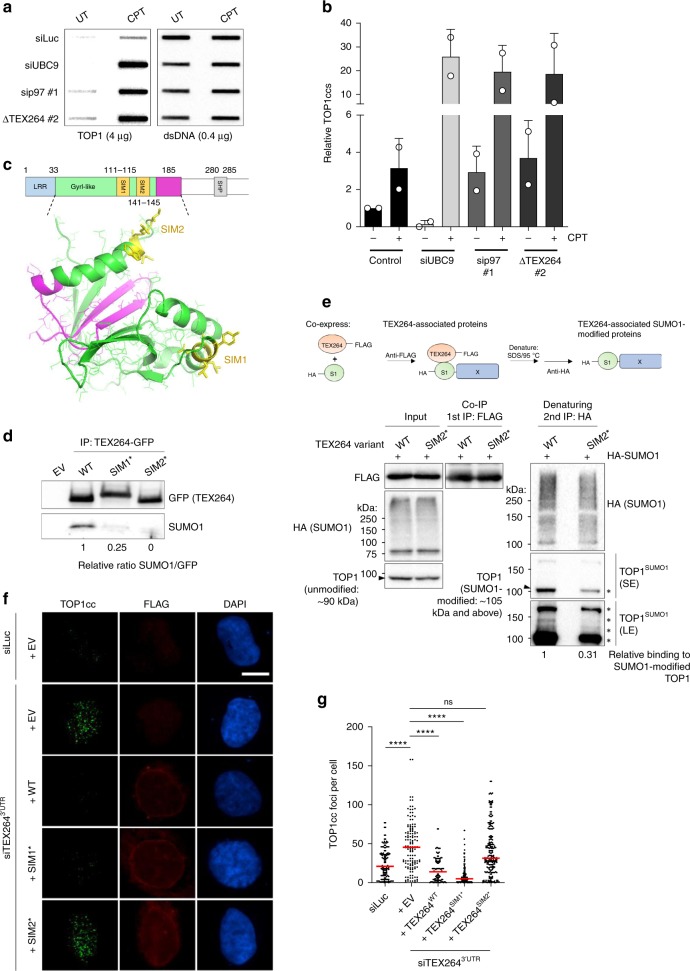
TEX264 can bind SUMO1-modified TOP1. **a** Analysis of TOP1ccs isolated by RADAR from the indicated cell lines. Where indicated, cells were treated with 1 μM CPT for 30 min. **b** Quantification of **a** (error bars represent mean ± SD; *n* *=* 2). **c** Above: schematic diagram of TEX264, indicating the location of its putative SUMO-interacting motifs (SIMs). Below: structural model of the GyrI-like domain of TEX264 generated using Phyre2. Residues 158–184 are highlighted in pink. Putative SIMs are highlighted in yellow. **d** Immunoblot of GFP & SUMO1 after incubation of purified GFP-tagged WT & SIM mutant TEX264 with free SUMO1. **e** Above: tandem-affinity purifications (TAP) procedure to identify SUMO1–modified proteins that interact with TEX264. Below: immunoblots of TAP experiment performed in ΔTEX264 HEK293 expressing the indicated variants of TEX264 and HA-SUMO1. SE and LE denote short and long exposure, respectively. Asterisks indicate SUMO1-modified versions of TOP1. **f** Immunofluorescent detection of TOP1ccs (green) and TEX264-FLAG (red) in U2OS cells transfected with the indicated siRNAs and cDNAs. Scale bar, 10 μm. **g** Quantification of experiments represented in **f**. Red line indicates median. Data are representative of two independent experiments. Statistical analysis was performed using Kruskal–Wallis ANOVA with multiple comparisons, with Dunn's post-test. Source data are available online.

We next examined the effects of TEX264 inactivation on TOP1cc repair in unchallenged conditions. Depletion of TEX264 led to significant TOP1cc foci accumulation in RPE-1 and U-2 osteosarcoma (U2OS) cells (Figs. 3a, b, 4f). Knockout of *TEX264* also caused substantial TOP1cc accumulation in HEK293 cells (Fig. 3c, d, Supplementary Fig. 3A). This was specifically due to loss of TEX264 as expression of exogenous TEX264 in ΔTEX264 cells could completely reverse TOP1cc accumulation (Supplementary Fig. 3B). Exogenous TEX264 could not reverse TOP1cc accumulation when ΔTEX264 cells were depleted of p97, indicating that TEX264 requires p97 to counteract TOP1cc accumulation (Supplementary Fig. 3B). Moreover, following a short CPT treatment and release into CPT-free media, TOP1ccs were rapidly resolved in control cells, but persisted long after CPT withdrawal in ΔTEX264 cells (Supplementary Fig. 3C, D). Together, these data demonstrate that TEX264 plays an important role in TOP1cc repair.

To test whether the increased levels of TOP1ccs in TEX264-deficient cells led to an increase in DNA damage, we performed an alkaline comet assay (Fig. 3e, f). Cells depleted of TEX264 displayed increased levels of basal DNA damage compared with control cells. Moreover, after CPT treatment, TEX264-depleted cells exhibited significantly delayed DNA damage repair kinetics—similar to TDP1-depleted cells. When TEX264 and TDP1 were co-depleted, there was no additive increase in DNA damage upon CPT treatment nor was there a further delay in DNA repair kinetics (Fig. 3f). This suggests that TEX264 and TDP1 act within the same TOP1cc repair pathway. To investigate further whether TEX264 and TDP1 are epistatic, we monitored TOP1ccs by RADAR. Depleting TEX264, TDP1, or p97, either alone or in pairs, resulted in similar increases in basal TOP1cc levels (Supplementary Fig. 3E–G). Moreover, TEX264- or TDP1-depleted HeLa cells are sensitive to low doses of CPT, and this is not enhanced by their co-depletion (Fig. 3g). ΔTEX264 HeLa cells were similarly sensitive to CPT (Supplementary Fig. 3H). Notably, overexpression of TEX264, but not TDP1, in TEX264-depleted cells reversed their sensitivity to CPT, indicating that TDP1 alone is not sufficient for TOP1cc repair (Supplementary Fig. 3I).

We next investigated the function of TEX264’s GyrI-like domain (Fig. 3h). The crystal structure of the bacterial GyrI protein, SbmC, revealed that the protein forms a solvent-exposed surface which, we inferred, may mediate substrate binding^49^. Based on this information, we generated TEX264 variants with single point mutations in conserved residues in or close to its GyrI-like domain and tested their ability to bind TOP1 (Fig. 3h, i, Supplementary Fig. 4A, B). Each variant displayed reduced binding to TOP1, but not p97, suggesting that they comprise a binding surface that enables TEX264 to bind TOP1 (Supplementary Fig. 4B). We focused on the E194A mutant since mutating this residue strongly reduced the TEX264–TOP1 interaction and because this residue resides in a predicted α-helix (amino acids 193–210) that is unique to TEX264 among GyrI-like proteins (Fig. 3i, Supplementary Fig. 4A & B). When TEX264 expression was suppressed using siRNA targetting its 3′UTR, U2OS cells accumulated ~2–3-fold more TOP1cc foci. Wild-type TEX264 could completely reverse this increase, whereas the TOP1-binding defective variant, E194A, largely failed to do so (Supplementary Fig. 4C, D). These results demonstrate that the interaction between TEX264 and TOP1 is important for TOP1cc repair. Altogether, our data suggest that TEX264 is a crucial TOP1cc repair factor that is epistatic with TDP1.

### TEX264 can bind SUMOylated TOP1

Most p97 cofactors have ubiquitin-binding domains that direct p97 to ubiquitinated proteins^37^. TEX264, however, does not appear to contain ubiquitin-binding motifs. As the ubiquitin-like protein, SUMO1, is proposed to facilitate TOP1cc repair, and Cdc48 has been shown to act on SUMOylated substrates, such as Rad52, we investigated whether TEX264 is linked to SUMO1-mediated TOP1cc repair^25,50–52^.

CPT treatment induced a dose-dependent increase in SUMO1-modified TOP1, indicating that TOP1ccs are SUMOylated (Supplementary Fig. 5A). We reasoned that SUMO1 might serve as an additional signal for the recruitment of TEX264. In the absence of TEX264 and p97, SUMO1-modified TOP1 would therefore be expected to accumulate. Indeed, depletion of either TEX264 or p97 caused SUMO1-modified TOP1 to accumulate (Supplementary Fig. 5B, C). We next sought to establish whether SUMOylation plays a role in TOP1cc repair. Consistent with previous reports, TOP1cc levels were initially lower when the E2 SUMO ligase, UBC9, was depleted (Fig. 4a, b)^53^. However, after CPT treatment, TOP1cc levels were strikingly higher in UBC9-depleted cells than in control cells and this was similar in extent to p97- or TEX264-inactivated cells (Fig. 4a, b). These data demonstrate that SUMOylation promotes the resolution of TOP1ccs, as previously reported, and suggest its importance for mediating TEX264 function in TOP1cc repair^3,51,52,54,55^.

Bioinformatic analysis subsequently revealed the presence of two putative SUMO-interacting motifs (SIMs) located in the GyrI-like domain of TEX264 (Fig. 4c). We therefore tested whether TEX264 directly interacts with SUMO. We found that TEX264 bound to free SUMO1, but not SUMO2 (neither free SUMO2 nor poly-SUMO2 chains; Fig. 4d, Supplementary Fig. 5D). Mutation of either of the putative SIMs strongly diminished SUMO1 binding, suggesting that both SIMs contribute to SUMO1 binding to some extent (Fig. 4d). Modified species of TOP1 co-immunoprecipitated less readily with the SIM2-mutated variant of TEX264 (TEX264^SIM2*^) than they did with either wild-type TEX264 (TEX264^WT^) or the SIM1-mutated variant (TEX264^SIM1*^; Supplementary Fig. 5E). To gain insight into why SIM2 appears to be more important for binding to modified species of TOP1, we generated a homology-based structural model of the GyrI-like domain of TEX264 (Fig. 4c). This revealed that SIM2 comes into closer proximity than SIM1 to the TOP1-binding surface (indicated in magenta in Fig. 4c) on TEX264.

To address specifically whether TEX264 interacts with SUMO1-modfied TOP1 in a SIM2-dependent manner, we performed tandem affinity purifications (TAP) from HEK293 cells co-expressing FLAG-tagged TEX264 and HA-tagged SUMO1 (Fig. 4e). We first purified TEX264–FLAG protein complexes under native conditions (1st IP). From here, we isolated HA-tagged SUMO1-modified interacting partners of TEX264 under denaturing conditions (2nd IP; Fig. 4e). This analysis revealed that many SUMOylated proteins exist in complex with TEX264^WT^ and, to a slightly lesser extent, with TEX264^SIM2*^ (Fig. 4e). When HA-SUMO1 eluates were probed specifically for TOP1, we observed that ~70% less SUMO1-modified TOP1 co-immunoprecipitated with TEX264^SIM2*^ than with TEX264^WT^ (Fig. 4e). These data demonstrate that TEX264 binds SUMO1-modified TOP1 and that this binding is strongly reduced when SIM2 is mutated.

In turn, TEX264^WT^ and TEX264^SIM1*^ could reverse TOP1cc accumulation in TEX264-depleted U2OS cells, whereas TEX264^SIM2*^ displayed a strongly reduced ability to do so (Fig. 4f, g). Overall, our results demonstrate that TEX264 can bind both SUMO1-modified and unmodified TOP1 (Figs. 2b, 4e, Supplementary Fig. 2D). We hypothesise that the TEX264–SUMO1 interaction may facilitate or stabilise the binding between TEX264 and TOP1ccs. In this way, TOP1 SUMOylation upon TOP1cc formation may enable TEX264 to be recruited more efficiently to TOP1ccs.

### TEX264 promotes SPRTN-dependent TOP1cc repair

In principle, TEX264 and p97 might together be capable of recognising and remodelling TOP1ccs to facilitate access of TDP1 to the phosphodiester bond that links TOP1 to DNA. Whether other factors contribute remained unclear. As p97 forms a homohexamer, it can bind multiple cofactors at a time^38,56^. It was recently demonstrated that another p97 cofactor, SPRTN, is a metalloprotease which can proteolytically cleave TOP1, amongst other DNA–protein crosslinks (DPCs), during DNA replication^18,19,57–60^.

**Fig. 5:**
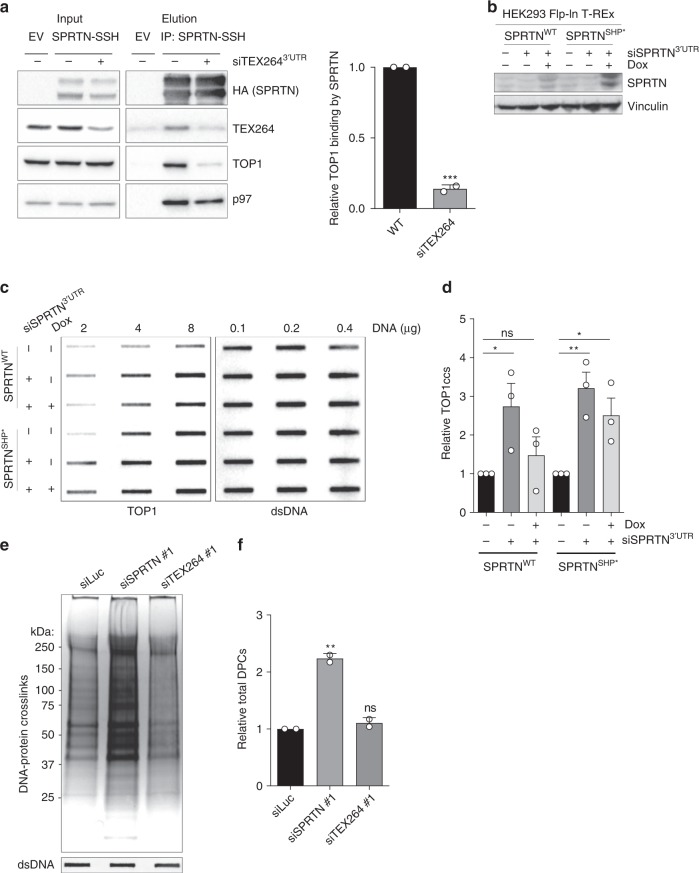
TEX264 cooperates with the metalloprotease SPRTN in TOP1cc repair. **a** Left: analysis of anti-Strep-tag immunoprecipitates prepared from HEK293 cells expressing SPRTN-SSH, transfected with the indicated siRNAs. Right: corresponding quantification of two independent experiments (mean ± SD; ***P < 0.0005). **b** Immunoblots to confirm SPRTN depletion/induction in doxycycline (Dox)-inducible HEK293 Flp-In T-REx cells used in c and **d**. Cells were transfected with siRNA targeting the 3′UTR of SPRTN 3 days prior to collection. Dox was added for the final 24 h. **c** TOP1cc detection by RADAR in same cells as **b**. **d** Quantification of **c** (error bars represent mean ± SEM; Student’s *t*-test; *n* = 3; *P < 0.05; **P < 0.01; ns, not significant). **e** Total DNA–protein crosslinks isolated by RADAR from HEK293 cells treated with the indicated siRNAs. DPCs were resolved by SDS–PAGE and visualised by silver staining. **f** Quantification of **e** (error bars represent mean ± SD; *n* *=* 2; **P < 0.01; ns, not significant; Student’s *t*-test). Source data are available online.

To assess the interplay of TEX264 and SPRTN in vivo, we depleted both proteins in HeLa cells, either alone or in combination, and assessed cellular sensitivity to CPT. Depletion of SPRTN alone sensitised cells to CPT, albeit to a lesser extent than TEX264 depletion (Supplementary Fig. 6A, B). However, co-depletion of SPRTN did not further sensitise TEX264-depleted cells to CPT, indicating that these proteins can co-operate to repair TOP1ccs but also that TEX264 has SPRTN-independent roles in counteracting TOP1ccs. To explore how TEX264 regulates SPRTN-dependent TOP1cc processing we immunoprecipitated SPRTN-SSH from wild-type and TEX264-depleted HEK293 cell extracts. Endogenous TOP1, p97, and TEX264 co-immunoprecipitated with SPRTN-SSH in wild-type cell extract (Fig. 5a). Depletion of TEX264 strongly reduced the interaction between SPRTN and TOP1 without affecting total TOP1 levels or the interaction between SPRTN and p97 (Fig. 5a). This indicates that TEX264 recruits p97–SPRTN sub-complexes to TOP1.

A recent structural study demonstrated that the protease active site of SPRTN is located within a narrow groove that is only accessible to flexible peptide structures and not globular proteins such as TOP1^61^. Therefore, large DPCs, such as TOP1ccs, likely need to undergo remodelling before their cleavage. Consistent with this, we previously demonstrated that SPRTN preferentially cleaves unstructured protein regions, most likely because they can more readily access SPRTN’s active site^19^. We therefore hypothesised that p97 might be needed to remodel TOP1ccs in order to make them more amenable to cleavage by SPRTN. To test this model, we performed an in vitro assay to assess the ability of purified SPRTN to process TOP1ccs isolated from cells by CsCl-gradient fractionation. SPRTN alone could process a small proportion of TOP1ccs. However, this activity was enhanced when TOP1ccs were pre-incubated with p97 or TEX264 and p97 prior to the addition of SPRTN (Supplementary Fig. 6C, D).

An implication of this model is that SPRTN would require its association with p97 to promote TOP1cc repair. To test this, we generated doxycycline-inducible stable cell lines expressing either wild-type SPRTN or a SPRTN point mutant that cannot bind p97 (SHP*; G255A, G257A, L260A, G261A; Fig. 5b). Wild-type SPRTN completely reversed TOP1cc accumulation in cells in which endogenous SPRTN was depleted with an siRNA sequence targetting its 3′UTR (Fig. 5c). However, the p97 binding-defective variant failed to do so, demonstrating that the SPRTN–p97 interaction is required for TOP1cc repair (Fig. 5c). Interestingly, we found that depletion of TEX264, unlike SPRTN, did not cause total DPCs to accumulate, suggesting that TEX264 may specifically promote SPRTN-dependent TOP1cc repair (Fig. 5e, f).

### TEX264 acts at replication forks

**Fig. 6:**
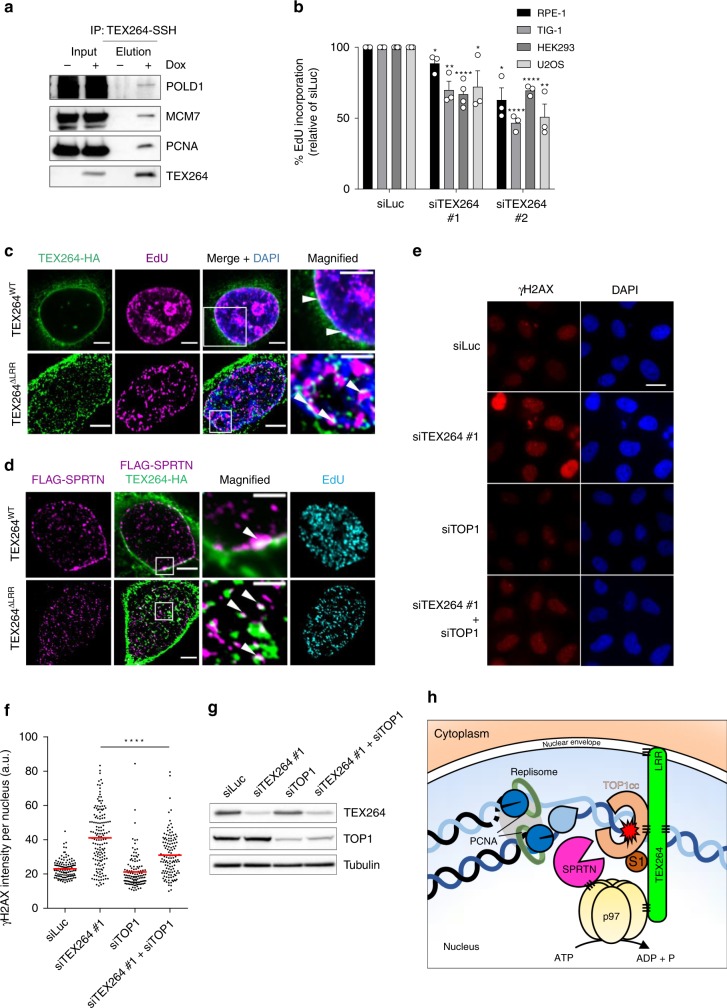
TEX264 acts at replication forks. **a** Immunoblots of anti-Strep-tag immunoprecipitates prepared from doxycycline (Dox)-inducible TEX264-SSH HEK293 Flp-In TRex cells, incubated with and without Dox. **b** Measurement of EdU incorporation rates in four different cell lines treated with the indicated siRNAs. EdU incorporation is plotted as a percentage of the corresponding control cells. Cells were incubated with 10 µM EdU for 30 min prior to collection (error bars represent mean ± SEM; ****P < 0.0001; ***P < 0.0005; **P < 0.01, *P < 0.05; Student’s *t*-test; experiments were performed at least three times). **c** Confocal images of U2OS cells transfected with the indicated cDNAs encoding SSH-tagged variants of TEX264 and stained with an anti-HA antibody. TEX264^ΔLRR^ image was deconvolved. Cells were treated with 10 µM EdU for 30 min before fixation. EdU was labelled using a Click-iT™ Alexa Fluor imaging kit. White arrowheads indicate examples of co-localisation. Scale bars, 5 μm (large panels) and 2 μm (magnified panels). See also Supplementary Fig. 7C. **d** Same as in **c**, except cells were co-transfected with FLAG-tagged SPRTN and co-stained with an anti-FLAG antibody. **e** Representative images of HeLa cells transfected with the indicated siRNA and stained with an antibody against γH2AX (phosphorylated on Ser139). **f** Quantification of the mean nuclear γH2AX intensity for experiment in **e** (****P < 0.0001; one-way ANOVA; red line indicates mean values). At least 100 nuclei were measured per condition. Scale bar, 20 μm. **g**, Immunoblots to confirm the efficacy of TEX264 and TOP1 depletions. **h** Proposed model: TEX264 is tethered at the nuclear periphery by its LRR. TEX264 binds to unmodified and SUMO1-modified TOP1 and counteracts TOP1cc accumulation by recruiting p97-SPRTN sub-complexes to TOP1ccs. S1 denotes SUMO1. Source data are available online.

SPRTN’s function is tightly coupled to DNA replication^19,24,60,62–64^. Based on this and the fact that TOP1ccs can stall DNA replication, we asked whether TEX264 acts near replication forks to prevent TOP1ccs from impeding fork progression. To identify interacting partners of TEX264, we isolated chromatin from doxycycline-inducible stable HEK293 cells expressing TEX264-SSH and analysed anti-Strep-tag immunoprecipitates by LC–MS/MS (Source Data). In addition to TOP1 and p97, we detected numerous replication factors, including PCNA and components of the MCM complex. We validated these interactions in co-immunoprecipitation experiments (Fig. 6a). Depletion of TEX264 with either of two different siRNA sequences in four different cell lines led to a significant reduction in EdU incorporation, which is suggestive of a defect in DNA replication (Fig. 6b). Furthermore, measurement of DNA replication fork velocity by DNA fibre assay revealed that depletion of TEX264 or p97 caused DNA replication forks to progress more slowly (Supplementary Fig. 7A).

We next investigated whether TEX264 localises to sites of DNA replication. Using confocal microscopy, we observed that TEX264 localises to the nuclear periphery, where it partially co-localises with newly synthesised DNA (i.e. EdU-positive DNA), SPRTN, and TOP1ccs (Fig. 6c, d; Supplementary Fig. 7B–D). A recent study demonstrated that the N-terminal LRR of TEX264 is a transmembrane segment that tethers it to the membrane of the ER^41^. When we deleted this segment (TEX264^ΔLRR^; amino acids 1–33), TEX264 redistributed into the cytoplasm as well as the nucleoplasm (Fig. 6c, d, Supplementary Fig. 7B). This suggests that a subset of TEX264 may be localised to the inner nuclear membrane via its LRR. Within the nucleus, TEX264^ΔLRR^ also partially co-localised with nascent DNA, SPRTN foci, and TOP1ccs (Fig. 6c, d, Supplementary Fig. 7D). Like some other GyrI-like proteins, recombinant TEX264 (purified without its LRR) binds DNA when analysed by electrophoretic mobility shift assay (EMSA; Supplementary Fig. 7E)^65^. These data indicate that the TEX264 protein has properties that enable it to associate with DNA, replication forks, TOP1ccs, and SPRTN, independently of its LRR/membrane localisation. Collectively, our data support a model in which TEX264 is present at the nuclear periphery where it can associate with SPRTN and DNA replication forks.

To biochemically demonstrate that TEX264 associates with DNA replication forks, we isolated newly replicated DNA from cells by isolation of proteins on nascent DNA (iPOND)^66^. Consistent with our microscopy data, TEX264 was detected, along with p97 and SPRTN, at/near replication forks by iPOND (Supplementary Fig. 7F). When cells were treated with CPT (100 nM, 1 h), we detected a striking increase in the amount of TOP1 at replication forks in ΔTEX264 cells compared with control cells (Supplementary Fig. 7F). This strongly supports the notion that TEX264 counteracts TOP1cc accumulation near replication forks (Fig. 6h). The amount of SPRTN detected at replication forks was slightly reduced in ΔTEX264 cells, suggesting TOP1ccs are common substrates of SPRTN during DNA replication (Supplementary Fig. 7F). In ΔTEX264 cells, p97 was still detected at DNA replication forks. This is likely because p97 has pleiotropic roles in DNA replication and can be recruited to replication forks by different p97 cofactors to, for example, regulate DNA replication origin firing or promote DNA replication termination by unloading the CMG helicase (Supplementary Fig. 7F)^67–71^.

Since replication-blocking TOP1ccs can induce DNA damage, we reasoned that reducing the prevalence of TOP1ccs should alleviate the DNA damage observed in TEX264-deficient cells. Strikingly, depletion of TOP1 strongly reduced DNA strand break accumulation in TEX264-depleted cells, as measured by γH2AX (Fig. 6e–g). Thus, the DNA strand breaks observed in TEX264-deficient cells can largely be attributed to the deleterious action of the TOP1 protein and the consequent formation of endogenous TOP1ccs.

## Discussion

TOP1ccs are highly cytotoxic endogenous DNA lesions that can also be exploited in cancer therapy. It is anticipated that targetting TOP1cc repair factors could enhance the clinical efficacy of TOP1 poisons and/or overcome drug resistance^1^. We have elucidated a key aspect of the TOP1cc repair process, specifically how TOP1ccs are processed upstream of the phosphodiesterase TDP1. The bulky nature of the TOP1 protein restricts TDP1’s access to the phosphodiester bond that links TOP1 to DNA. Heat denaturation or pre-digestion of TOP1ccs with trypsin enables TDP1 activity in vitro, however, a detailed understanding of TOP1cc processing upstream of TDP1 in vivo has been lacking^11–13^.

Here we report that TEX264 acts with the p97 ATPase and SPRTN metalloprotease to repair TOP1ccs. We found that TEX264 is recruited to TOP1ccs and binds the TOP1 protein via residues clustered in and around its GyrI-like domain. TEX264 also possesses SIMs that enable it to interact with SUMO1-modified TOP1. TEX264 interacts with p97 and SPRTN and bridges their interactions with TOP1. Our data demonstrate that the ATPase activity of p97 is required for TOP1cc repair and suggest that p97 facilitates the proteolytic digestion of TOP1ccs by SPRTN. The removal of the bulk of the protein component of a TOP1cc could then enable TDP1 to excise the remnant DNA-bound peptide^12^.

Two recent studies reported that TEX264 is embedded in the ER membrane via its transmembrane N-terminal LRR, where it functions as an ER-phagy receptor^41,42^. Our finding that deleting the LRR of TEX264 causes it to mobilise into the nucleoplasm suggests that a subset of TEX264 is localised at the inner nuclear membrane. TOP1 has recently been shown to predominantly act on heterochromatin that is tethered to the nuclear envelope, presumably because these genomic regions are most prone to topological disruptions^72^. It is therefore plausible that TOP1ccs may be most prevalent in the vicinity of the nuclear envelope. This is a particularly appealing model considering that TEX264 and SPRTN co-localise with nascent DNA at the nuclear envelope, where heterochromatin is tethered. Alternatively, TOP1ccs may be relocalised to the nuclear envelope, as has been demonstrated for other SUMOylated proteins and some types of DNA lesions^73–75^. It is perhaps less likely that TEX264 only operates on a specific subset of TOP1ccs, since numerous experimental approaches demonstrated that TEX264 and TDP1 act in the same pathway to repair endogenous TOP1ccs. Our data therefore suggest that TOP1cc repair may occur at the nuclear periphery. There may otherwise be a mechanism of releasing TEX264 from the nuclear envelope or alternative TEX264 isoforms which lack the LRR.

TEX264 can also bind the autophagosomal protein LC3B^41,42^. In line with our findings, proteomic analyses conducted by An et al. (2019) also identified TOP1 and VCP/p97 as highly abundant TEX264-interacting partners, amongst numerous other nuclear proteins. Whether TEX264’s role in autophagy is linked to the observations presented here is an interesting line of future enquiry. The SUMO system, Cdc48/p97, and autophagy have previously been linked to DPC repair in yeast^17^. The Cdc48 cofactor, Wss1, relocalises to vacuoles in response to genotoxic stress and this is proposed to promote the clearance of TOP1ccs via autophagy^17^.

In addition to Wss1, the Cdc48 cofactor, Ufd1, possesses a SIM which is important for TOP1cc repair in yeast^25^. While the same SIM in yeast Ufd1 is not conserved in humans, we do not rule out a role for Ufd1 in TOP1cc repair in human cells. For instance, p97 hexamers can bind cofactors in a hierarchical manner, as described for Ufd-Npl4 and FAF1, which can provide an additonal layer of substrate-specificity control^56^.

The function of Wss1 in TOP1cc repair depends on Cdc48 but the reasons for this were unclear^16,17^. It was speculated that Cdc48 could be involved in the removal of peptide remnants generated by proteolysis or in remodelling the TOP1 protein to facilitate its proteolytic digestion^16^. Our data suggest that the p97–TEX264 complex enables TOP1cc proteolysis by SPRTN. This conclusion is supported by a recent strutural analysis of SPRTN’s active site^61^. The catalytic core of SPRTN is located within a narrow groove that is only accessible to small, flexible peptides, such as the disordered tails of histones. By contrast, TOP1ccs have a large, globular structure and lack flexible linker regions that could be accommodated by the substrate-binding groove of SPRTN. Therefore, the requirement of TEX264 and p97 in TOP1cc repair likely reflects the need to recognise and remodel the TOP1 protein to render it more amenable to cleavage by SPRTN. The fact that TEX264 appears to be dispensable for the clearance of the majority of DPCs supports the existence of a specialised TOP1cc repair complex consisting of TEX264, p97, and SPRTN.

It will be interesting to explore whether this mode of SPRTN recruitment is more widely applicable to other SPRTN substrates, since little is known about how SPRTN is recruited to, and recognises, specific DPCs. SPRTN can be recruited to stalled replication forks by binding to PCNA (via its PIP box) or ubiquitinated proteins (via its UBZ)^24,62,76–78^. However, some recent evidence indicates that these domains are not essential for the recruitment of SPRTN to chromatin in response to DPC formation nor for DPC repair^18,20,59,79^.

Finally, our findings raise the possibility that the p97 system could be targetted to counteract clinical resistance to CPT.

## Methods

### Cell culture

Human HEK293, U2OS, RPE-1, and HeLa cells were obtained from ATCC. All cell lines were cultured in DMEM containing 10% FBS and 5% Penicillin/Streptomycin. All cell lines were regularly screened for mycoplasma using a MycoAlert™ Mycoplasma Detection Kit.

### Generation of cell lines

To generate CRISPR–Cas9 TEX264 knockout cells, two plasmids—a CRISPR/Cas9 KO plasmid containing guide RNA targetting TEX264 (sc-417333), and a homology-directed repair plasmid containing a puromycin resistance cassette (sc-417333-HDR)—were purchased from Santa Cruz. 2.5 μg of each plasmid was transfected into early-passage HEK293 and HeLa cells using Fugene HD (Promega). After 72 h, media supplemented with puromycin was added to the cells. The puromycin dose required to kill wild-type cells was determined to be: 1.25 μg/mL for HEK293 cells and 0.6 μg/mL for HeLa cells. After 72 h, the puromycin-containing media was removed and cells were sorted using a cell sorter into single-cell populations on a 96-well plate. TEX264 expression was analysed by immunoblotting. Multiple clones of each cell line showing loss of all detectable TEX264 were selected for subsequent analysis.

Flp-In™ T-REx™-293 cells (ThermoFisher Scientific) were cultured in DMEM complete media containing Zeocin (100 µg/mL; ThermoFisher Scientific) and Blasticidin (15 µg/mL; Cambridge Biosciences). TEX264 or SPRTN-SHP* cDNA was cloned into the pCDNA5/FRT/TO-cSSH vector. Cells were grown in complete media supplemented with Blasticidin (15 µg/mL) and co-transfected with pCDNA5‐FRT/TO-TEX264-SSH or pCDNA5‐FRT/TO-SPRTN-SHP*-SSH and the Flp recombinase expression plasmid, pOG44 (ThermoFisher Scientific). After 2 days, single colonies were selected and expanded in DMEM complete media containing Blasticidin (15 µg/mL) and Hygromycin B (50 µg/mL; Caymen Chemical). Clones were then tested for TEX264-SSH or SPRTN-SHP*-SSH expression upon induction with 1–1.2 µg/mL Doxycycline by immunoblotting. Flp-In T-Rex-293 cells expressing miRNAs targetting TDP1 were generated as previously described^80^.

### Cellular fractionations

The cytoplasmic fraction was obtained by resuspending cells in buffer A (10 mM HEPES, 10 mM KCl, 340 mM sucrose, 10% glycerol, protease and phosphatase Inhibitors, and 2 mM EDTA). Triton X-100 was added to a final concentration of 0.1% and cells were incubated on ice for 5 min. Cells were then centrifuged at 350×*g* for 3 min. The remaining pellet (nuclei) was washed in buffer A without Triton X-100. Nuclei were ruptured by resuspending in buffer B (3 mM EDTA, 0.2 mM EGTA, 5 mM HEPES pH 7.9, protease and phosphatase inhibitors). The nuclear fraction was then centrifuged at 1700×*g* for 5 min and the supernatant was collected as the nuclear soluble fraction. The chromatin pellet was washed twice in Benzonase buffer (50 mM Tris–HCl pH 7.9, 50 mM NaCl, 5 mM KCl, 3 mM MgCl_2_, and protease and phosphatase inhibitors) and digested in Benzonase buffer, supplemented with 125 U of Benzonase (Merk Millipore) either at room temperature for 30 min or overnight rolling at 15 rpm at 4 °C. Samples were then centrifuged at 20,000×*g* for 5 min, the supernatant was collected as the chromatin soluble fraction.

### Immunoprecipitation

Cells were lysed in IP lysis buffer (50 mM Tris, pH 7.4, 150 mM NaCl, 1 mM EDTA, 0.5% Triton X-100, supplemented with protease and phosphatase inhibitors) and incubated on a rotator at 4 °C for 10 min. Chromatin was then pelleted by centrifugation for 5 min at 1000×*g* and then digested with 100 U/mL Benzonase at room temperature in Benzonase buffer (2 mM MgCl_2_, 50 mM Tris, pH 7.4, 150 mM NaCl). For denaturing IPs, samples were supplemented with SDS (final concentration 1%) and incubated on ice for 10 min. Denaturing IPs were quenched with Triton X-100 (to a final concentration of 1%). Lysates were supplemented with ethidium bromide (50 μg/mL) and incubated with anti-FLAG M2 agarose (Sigma-Aldrich), Strep-Tactin Sepharose (IBA), or GFP-Trap agarose (Chromotek) for 1–3 h(s) on a rotator at 4 °C, washed three times with IP wash buffer (150 mM NaCl, 50 mM Tris–HCl; 0.5 mM EDTA), and resuspended in 2 × Laemmli buffer. For TAP experiments, cells were grown on 245 × 245 mm cell culture dishes (two per condition). Immunoprecipitation of TEX264-FLAG was performed as described above. After TEX264-FLAG was captured, samples were boiled for 5 min in 1% SDS then quenched with 1% triton and incubated with anti-HA beads (Thermo Fisher) for 16 h at 4 °C with rotation. The next day samples were washed three times in IP wash buffer and eluted in 2× Laemmlli buffer.

### Mass spectrometry sample preparation and analysis

YFP-TOP1: HEK293 cells were transfected with cDNA encoding YFP-tagged TOP1. After 48 h, chromatin was isolated from these cells as described above. Samples were incubated with GFP-Trap agarose for 3 h. Proteins were eluted using Laemmli buffer. Samples were reduced (5 mM DTT final concentration) and alkylated (20 mM iodacetimide final concentration) for 30 min at room temperature (each step) prior to performing a double methanol and chloroform protein precipitation to remove SDS. Protein pellets were solubilised with 8 M Urea in 20 mM HEPES (pH 8) and digested with immobilised trypsin for 16 h at 37 °C (Pierce; Urea final concentration < 1 M) following the standard in-solution digest protocols. The resulting tryptic peptides were then purified by solid phase extraction using SOLA HRP SEP cartridges (ThermoFisher) and dried^81^.

Ten percent of the tryptic peptides were analysed using a nano LC–MS/MS workflow based on a Dionex Ultimate 3000 UPLC connected to an Orbitrap Fusion Lumos (both Thermo Instruments). Peptides were separated on an EASY-Spray column (500 mm × 75 μm) over one hour gradient of 2–35% acetonitrile in 5% dimethyl sulfoxide/0.1% formic acid (flow rate of 250 nl/min) and analysed on the Orbitrap Fusion Lumos using the universal method (MS1 in the Orbitrap at 120k resolution, AGC target of 4e5; MS/MS: ion trap rapid scan mode, CID fragmentation at 35% collision energy, AGC target of 4.0E3 for up to 250 ms).

TEX264-SSH: Flp-In TRex HEK293 cells inducibly expressing SSH-tagged TEX264 were either left untreated or incubated overnight with 1.2 µg/mL Doxycycline. Cells were lysed in a buffer composed of 20 mM HEPES pH 7.45, 10 mM KCl, 137 mM NaCl, 0.25% digitonin, 20 mM NEM, 5 mM EDTA, and protease and phosphatase inhibitors. The chromatin pellet was washed once in lysis buffer supplemented with 0.1% Triton X-100, and three times in Benzonase buffer (25 mM NaCl, 25 mM Tris–HCl pH 7.9, 5 mM KCl, 2 mM MgCl_2_, supplemented with protease and phosphatase inhibitors). Chromatin was digested in buffer supplemented with 125 U Benzonase overnight at 4 °C on a rotating wheel. Samples were incubated with Strep-Tactin Sepharose for 2 h with rotation at 4 °C, after which the beads were washed three times in a buffer composed of 50 mM NaCl, 5 mM KCl, 50 mM Tris–HCl pH 7.5, 0.025% NP-40. Proteins were eluted by adding 2.5 mM biotin in 1x IP buffer and shaking the samples for 15 min at 4 °C. The samples were processed for MS by in solution digestion. In brief, the samples were reduced by addition of DTT to a final concentration of 5 mM and incubated for 30 min at room temperature. Iodoacetic acid (IAA) was then added to prevent to reformation of disulphide bridges to a final concentration of 20 mM and incubated for 30 min at room temperature. The proteins were then precipitated by the addition of methanol and chloroform. The pellet was then resuspended in 6 M Urea and sonicated for 2 min using the Bioruptor (5 s on, 5 s off for 10 cycles) to solubilise the protein. The samples were then diluted with ddH_2_O to dilute the Urea to below 1 M. Trypsin was then added to a concentration of 200 ng/µL and incubated at 37 °C overnight. The following morning, formic acid was added to a final concentration of 0.1% to decrease the pH and prevent further trypsin digestion. The protein peptides were then purified using a SEP-PAK column and run on an OrbiTrap LTQ Velos Elite Mass Spectrometer (30k resolution, top 20, collision induced dissociation)^82^.

#### Data analysis

Peptides and proteins were identified by searching the MS raw files against the Human SwissProt database downloaded in June 2017 (containing 20,206 sequences) for YFP-TOP1 and in February 2015 (20,274 sequences) for TEX264-SSH. MASCOT data outputs were filtered by applying a 20 ion cut off and 1% FDR above identity or homology threshold. The mass spectrometry proteomics data have been deposited to the ProteomeXchange Consortium via the PRIDE partner repository with the dataset identifier PXD017239 and 10.6019/PXD017239^83^.

### Protein purification and in vitro interaction assays

SPRTN was purified as previously described^19^. p97 was cloned into a pET21a vector (original vector pCDNA3.1 p97-S-tag was a gift from J. Christianson) and purified with a C-terminal S-Tag from Rosetta *E. coli* with S-protein Agarose (Merck Milipore) using standard methods. TEX264 was purified without its hydrophobic N-terminus (amino acids 1-25; ΔNT) to render the protein more soluble and enable purification. ΔNT TEX264 was cloned into a pET21a vector (with a C-terminal His-tag) and expressed in Rosetta *E. coli*. Cells were grown at 37 °C and induced for 6 h with 1 mM IPTG upon reaching an OD_600_ of 0.6. For ΔNT TEX264 the temperature was dropped to 21 °C, whereas for ΔNT TEX264^ΔSHP^ and p97 the temperature was kept at 37 °C. Cultures were centrifuged at 5000×*g* for 30 min and the pellets frozen at −80 °C overnight. The thawed pellets were resuspended in resuspension buffer (800 mM NaCl, 20 mM Tris–HCl pH 7.0) containing 1% Triton X-100 and 1 mM PMSF. Lysozyme was added and incubated on ice for 30 min followed by sonication (two minutes 20% power, 50% pulsing, Ultrasonic Homogeniser Model 300V/T, Biologics Inc.) and centrifugation at 20,000×*g* for 30 min. Ni-NTA agarose (Qiagen) pre-washed with resuspension buffer was incubated with the lysate at 4 °C for 2 h with rolling at 15 rpm. This was centrifuged at 500×*g* for 5 min and the supernatant removed. Beads were washed twice with resuspension buffer for 15 min rolling at 15 rpm at 4 °C (once with resuspension buffer supplemented with 50 mM Imidazole and then with 100 mM Imidazole). Proteins were eluted from the beads using resuspension buffer with a gradient of 200–1 M Imidazole. The fractions were analysed by SDS–PAGE and Coomassie (Instant blue, Expedeon) and pooled accordingly. The buffer was exchanged three times for storage buffer (150 mM NaCl, 10 mM Tris–HCl pH 7.5, 0.1 mM EDTA, 15% Glycerol) using EMD Millipore Amicon™ Ultra-15 Centrifugal Filter Units (Fisher Scientific) and subsequently stored at −80 °C.

Protein interaction studies were performed as follows: p97 was coupled to S-protein Agarose for 2 h at 4 °C in buffer containing 137 mM NaCl, 20 mM Tris–HCl pH 7.0, then incubated for 2 h with TEX264^WT^, TEX264^ΔSHP^, and/or recombinant TOP1 (Inspiralis) in the same buffer, supplemented with 0.5% Triton X-100. TEX264^WT^ was coupled to HisPur™ Ni-NTA Magnetic Beads (Thermo Fisher), prior to incubation for 2 h with recombinant TOP1 in a buffer composed of 137 mM NaCl and 20 mM Tris–HCl pH 7.0. Reactions were terminated by the addition of Laemmli buffer and resolved by SDS–PAGE.

### DNA-binding assay

DNA 5′ IRDye800-labelled probes (Integrated DNA Technologies) were either used as single stranded (ATTCGATCGGGGCGGGGCGAGC) or double stranded (annealed to a complementary unlabelled strand (GCTCGCCCCGCCCCGATCGAAT; Eurogentec) templates. The probes were incubated with BSA, Sp1 (gift from G. Dianov), or TEX264^WT^, as indicated, in protein storage buffer (150 mM NaCl, 10 mM Tris–HCl pH 7.5, 0.1 mM EDTA, 15% Glycerol) for 15 min at 37 °C. Samples were resolved on a 0.5% agarose gel in TBE at 4 °C before visualisation using the Odyssey image analysis system (Li-Cor Biosciences).

### Rapid approach to DNA adduct recovery

DPCs were isolated using a modified RADAR assay^34^. Cells were grown to ~70% confluency then lysed in M buffer (MB), containing 6 M guanidine thiocyanate, 10 mM Tris–HCl (pH 6.8), 20 mM EDTA, 4% Triton X-100, 1% N-lauroylsarcosine and 1% dithiothreitol. DNA was precipitated by adding 100% ethanol, then washed three times in wash buffer (20 mM Tris–HCl pH 7.4, 50 mM NaCl, 1 mM EDTA, and 50% ethanol), and solubilised in 8 mM NaOH. DNA concentrations were quantified by NanoDrop^TM^ and confirmed by slot blot analysis on a Hybond N + membrane followed by detection with an anti-dsDNA antibody. For TOP1ccs, samples were digested with Benzonase for 30 min at 37 °C and analysed by slot blotting on a Nitrocellulose membrane.

### Colony forming assay

HeLa cells were seeded in triplicate on six-well plates and allowed to attach for 16 h. Cells were then treated with the indicated doses of CPT (Sigma-Aldrich) for 24 h, after which they were washed with PBS, and cultured in DMEM complete media. Colonies were fixed and stained 7–10 days later and the number of colonies was counted using the automated colony counter, GelCount™ (DTI-Biotech). The number of colonies in treated samples is expressed as a percentage of the number of colonies in the untreated samples. Where indicated, cells were transfected with siRNA and plasmid DNA (2 µg DNA; FuGENE HD) 72 and 48 h, respectively, before CPT treatment.

### Alkaline comet assay

Cells were trypsinised, inactivated in 1% FBS-PBS, resuspended in 1% low melting point agarose in PBS (37 °C), and embedded on microscope slides pre-coated with 1% normal melting point agarose in dH_2_O. Cells were lysed for 1 h at 4 °C in a buffer containing 2.5 M NaCl, 100 mM EDTA, 10 mM Tris–HCl, 1% (v/v) DMSO and 1% (v/v) Triton X-100, at pH 10.5. Slides were then incubated in 4 °C electrophoresis buffer (300 mM NaOH, 1 mM EDTA, 1% (v/v) DMSO, pH > 13) for 30 min to unwind DNA. Electrophoresis was performed in the same buffer for 25 min at 25 V (300 mA). Cells were then neutralised in 0.5 M Tris–HCl pH 8.1 and stained with SYBR Gold (1:10,000 in dH_2_O) for 30 min. Microscopy and analysis were performed using the Nikon NiE microscope and Andor Komet7.1 software. At least 100 cells were quantified per condition. Data are shown as Olive tail moments, calculated as: (tail mean−head mean)*Tail%DNA/100.

### SUMO-binding assay

ΔTEX264 HEK293 cells transiently expressing GFP-tagged or FLAG-tagged TEX264 variants were lysed in a lysis buffer containing 50 mM Tris, pH 7.4, 500 mM NaCl, 1 mM EDTA, and 3% Triton X-100. Samples were sonicated using a Bioruptor Plus sonicator (30 s ON, 30 s OFF for three cycles), then diluted (1:3 volume) in IP wash buffer (150 mM NaCl, 50 mM Tris–HCl; 0.5 mM EDTA) and incubated with anti-FLAG M2 agarose or GFP trap beads (Chromotek) for 2 h. After capture, the beads were washed three times in IP wash buffer and incubated with 1 μg of free SUMO1 or SUMO2 (Boston Biochem) for 90 min at 4 °C with rotation. After washing three times in IP wash buffer, the samples were eluted in Laemmli buffer for 5 min at 95 °C.

### Isolation of proteins on nascent DNA

iPOND was performed as described previously^66^. Briefly, HEK293 cells were incubated with 10 μM EdU. For Supplementary Fig. 7F, EdU incubation times were 8 and 16 min for WT cells and ΔTEX264 cells, respectively, to account for the reduced EdU incorporation rates in ΔTEX264 cells. Where indicated, cells were treated with CPT (100 nM) for 1 h in normal media which was then supplemented with 10 μM EdU for the final 8 or 16 min. Chromatin was fragmented into 50–300 bp fragments by sonication with a Bioruptor Plus sonicator (30 s ON, 30 s OFF for 50 cycles). Biotin-labelled EdU was captured by incubating samples overnight with streptavidin-coupled agarose beads (Merck Millipore).

### DNA fibre assay

HEK293 cells were incubated in media containing 30 µM CldU (Sigma-Aldrich, C6891) for 30 min, washed 3× in 37 °C PBS, and then incubated with media containing 250 µM of IdU (Sigma-Aldrich, 17125) for an additional 30 min. Cells were then treated with ice-cold PBS. Cells were lysed in 200 mM Tris–HCl pH 7.4, 50 mM EDTA, and 0.5% SDS directly onto glass slides and then fixed with 3:1 methanol and acetic acid overnight at 4 °C. The next day, the DNA fibres were denatured with 2.5 M HCl, blocked with 2% BSA and stained with antibodies that specifically recognise either CldU (Abcam, Ab6326, dilution 1:500) or IdU (BD-347580, dilution 1:500). Anti-rat Cy3 (dilution 1:300, Jackson Immuno Research, 712-116-153) and anti-mouse Alexa-488 (dilution 1:300, Molecular Probes, A11001) were used as the respective secondary antibodies. Microscopy was performed using a Nikon 90i microscope. The lengths of the IdU-labelled tracts were measured using ImageJ software and converted into microns. Statistical analysis was performed using GraphPad Prism software using one-way ANOVA.

### Immunofluorescence

Cells were seeded on glass coverslips in six-well plates. Cells were fixed in 4% formaldehyde for 15 min at room temperature. Fixed cells were washed with 1× PBS and permeabilised in 0.5% Triton X-100 prepared in PBS for 15 min at 4 °C. After blocking in 5% BSA/PBS for 1 h at room temperature, cells were incubated with primary antibodies diluted in 2.5% BSA PBS solution for 1 h at room temperature. Coverslips were then washed three times in 1× PBS and incubated with secondary antibodies and DAPI (1:500) for 1 h at room temperature. Coverslips were mounted onto slides using ProLong Gold antifade reagent (Invitrogen) and imaged using a Nikon 90i microscope. For EdU staining, cells were treated with EdU (10 μM) for 30 min before fixing, and then stained using the Click-iT Plus EdU Alexa Fluor 647 imaging kit (Invitrogen). Confocal microscopy was performed using Andor Dragonfly 200 on a Nikon Ti-E microscope. Z-stacks were taken at an interval of 0.1 µM across cells fixed identically to normal brightfield microscopy. ClearView-GPU™ deconvolution was carried out on samples where stated. Images were imported via Bio-Formats (ImageJ plugin) and analysis was carried out using ImageJ. Images displayed are single z-slices unless otherwise stated.

Visualisation of TOP1ccs by immunofluorescence was performed essentially as previously described^36^. Briefly, cells were fixed in 4% formaldehyde for 15 min at room temperature, then permeabilised in 0.5% Triton X-100 for 15 min at 4 °C. Cells were then treated with 0.5% SDS for 5 min at room temperature and washed five times in a buffer containing 0.1% Triton X-100 and 0.1% BSA diluted in PBS. After blocking in 5% BSA/PBS for 1 h at room temperature, cells were incubated with an anti-TOP1cc antibody (Merck) diluted 1:100 in 2.5% BSA/PBS. Following staining with secondary antibodies and DAPI, coverslips were mounted onto slides using ProLong Gold antifade reagent (Invitrogen) and imaged using a Nikon 90i microscope. Where indicated, siRNA transfections were performed 72 h before cells were fixed. Plasmid transfections (1 µg plasmid DNA) were performed using FuGENE HD Transfection Reagent (Promega) 24 h before cell fixation.

### In vitro TOP1cc repair assay

FlpIn T-REx HEK293 cells inducibly expressing TDP1-targetting miRNAs were treated with 1 µg/mL doxycycline for 48 h to deplete TDP1, then treated with 10 μM MG132 for 1 h and 14 μM CPT for 30 min to generate stable TOP1ccs. Cells were lysed in a guanidine hydrochloride-based lysis buffer (8 M guanidine hydrochloride, 30 mM Tris–HCl pH 7.5, 10 mM EDTA, 1% sarkosyl, pH adjusted to 7.5) and separated by CsCl-gradient fractionation at room temperature for 24 h^84^. TOP1ccs fractions were pooled and precipitated with ice cold 100% acetone at −80 °C for 30 min, washed with 70% ethanol, air dried, and resuspended in dialysis buffer (50 mM HEPES–KOH pH 7.5, 50 mM NaCl, 10 mM MgCl_2_, 0.5 mM TCEP) supplemented with protease inhibitors. Samples were dialysed using Slide-A-Lyzer cassettes in 100× dialysis volume overnight at 4 °C. Samples were sedimented by centrifugation at 15,000×*g* for 20 min to remove aggregated proteins. Samples were incubated with recombinant BSA, p97, and/or TEX264 (100 nM/reaction) in reaction buffer (50 mM HEPES pH 7.5, 50 mM NaCl, 10 mM MgCl_2_, 0.5 mM TCEP, ATP, 0.1 mg/mL protease-free BSA) at 37 °C for 30 min, before addition of the indicated concentrations of recombinant SPRTN for 1 h. Samples were then slot blotted onto a nitrocellulose membrane, blocked with Odyssey blocking buffer (LI-COR) at room temperature for 1 h, and probed with a TOP1cc-specific antibody at 4 °C overnight. After 3× TBS-T wash, the membrane was probed with IRDye 680RD goat anti-mouse secondary antibody (LI-COR) at room temperature for 1 h. After 3× TBS-T wash, the membrane was imaged using Odyssey Fc Imaging system (LI-COR).

### Flow cytometry

Seventy-two hours after siRNA transfection, cells were incubated with EdU (Thermo Fisher) at a final concentration of 10 µM for 30 min. Cells were collected, washed twice in 1× PBS, resuspended in 4% PFA and left for 15 min. Cells were then washed in 1× PBS, resuspended in FACS buffer (0.25% Saponin, 1% FBS in 1× PBS) by vortexing, and incubated for 15 min. The Click reaction carried out according to the manufacturer’s instructions using the Click-iT^®^ EdU Alexa Fluor^®^ 647 imaging kit (Thermo Fisher). Cells were washed once in FACS buffer before resuspension in 1% BSA (diluted in 1× PBS) containing 10 µg/µL RNAse and 20 µg/mL Propidium Iodide and incubated for 30 min. Samples were then run on the FACSCalibur (BD Biosciences) and analysed using FlowJo^®^. Representative gating strategy is provided as Supplementary Fig. 8.

### Site-directed mutagenesis

Site-directed mutagenesis was performed by PCR using the AccuPrime™ Pfx DNA Polymerase (ThermoFisher Scientific) according to the manufacturer’s instructions. For primer sequences, see Supplementary Table 1. Plasmids sequences were verified by sequencing at Source BioScience, Oxford, UK.

### Plasmid and siRNA Transfections

Plasmid DNA transfections were performed using polyethyleneimine (PEI) reagent, Lipofectamine 3000 (Thermo Fisher), or FuGENE HD Transfection Reagent (Promega), following the manufacturer’s instructions.

All siRNA transfections were carried out using Lipofectamine RNAiMAX (Invitrogen), according to the manufacturer’s protocol and assayed after 72 h.

The siRNA sequences used in this study are listed in Supplementary Table 2.

### Antibodies

The details of antibodies used in this study are provided in Supplementary Table 3.

### Reporting summary

Further information on research design is available in the Nature Research Reporting Summary linked to this article.

## Supplementary information

Supplementary Information

Reporting Summary

## Data Availability

The mass spectrometry raw datasets are publicly available through the ProteomeXchange Consortium via the PRIDE partner repository with the dataset identifier PXD017239 and 10.6019/PXD017239. The mass spectrometry datasets are also available in the Source Data file. Source data for Figs. 1–6 and Supplementary Figs. 1–7 are provided as a Source Data file. All other data are available from the corresponding author upon reasonable request.

## Source data

Source Data
