# Supplementary material for: TEX264 coordinates p97- and SPRTN-mediated resolution of topoisomerase 1-DNA adducts
Source: Nat Commun. 2020 Mar 9;11:1274. doi: 10.1038/s41467-020-15000-w (PMC7062751; doi:10.1038/s41467-020-15000-w)
Supplement: Supplementary file 1 — Supplementary Information [file 41467_2020_15000_MOESM1_ESM.pdf]

Supplementary information for

TEX264 coordinates p97- and SPRTN-mediated resolution of topoisomerase 1-DNA adducts

Fielden, Wiseman et al.

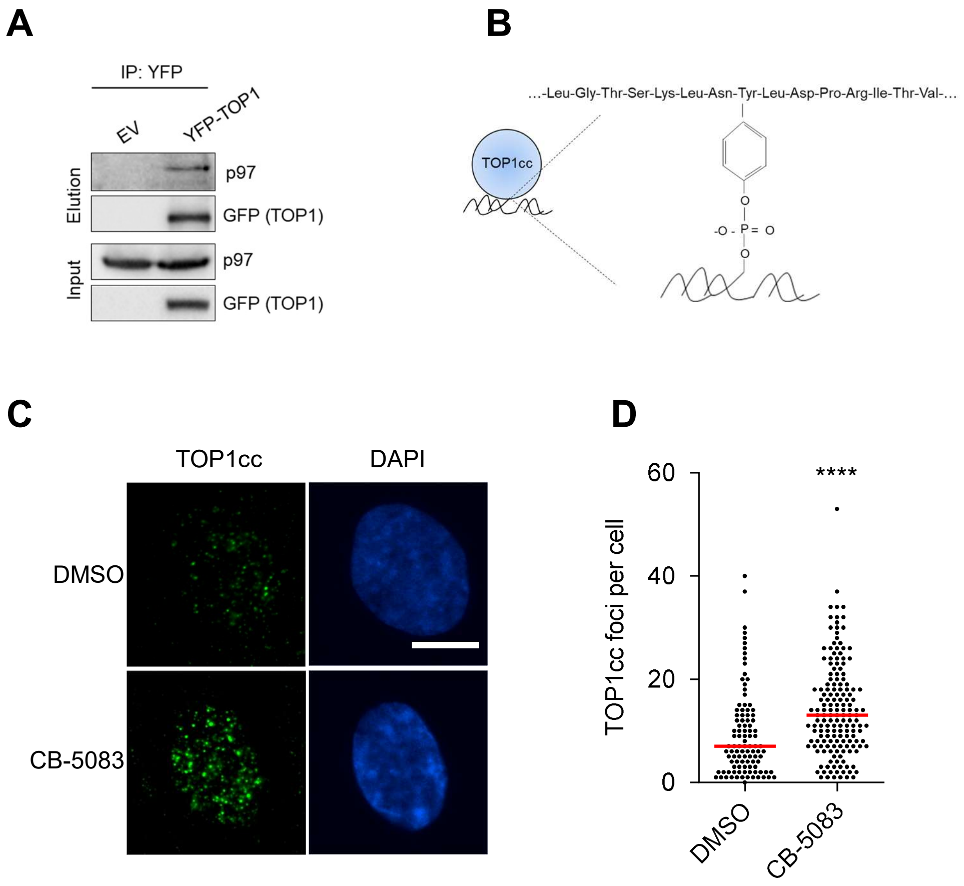

Supplementary Figure 1

Supplementary Figure 1: The p97 ATPase Promotes TOP1cc Repair

A, Co-immunoprecipitation of p97 with chromatin-bound YFP-TOP1. **B,** Schematic diagram of the antigen recognised by the TOP1cc-specific antibody. **C,** Representative nuclei of RPE-1 cells, treated with either DMSO or the p97 inhibitor, CB-5083 (10 μM), for 6 hours, then immunostained with the TOP1cc-specific antibody. Scale bar, 10 μm. **D,** Quantification of C. Red line indicates median values. Quantification of two experiments. Significance determined by Mann–Whitney test. Source data are available online.

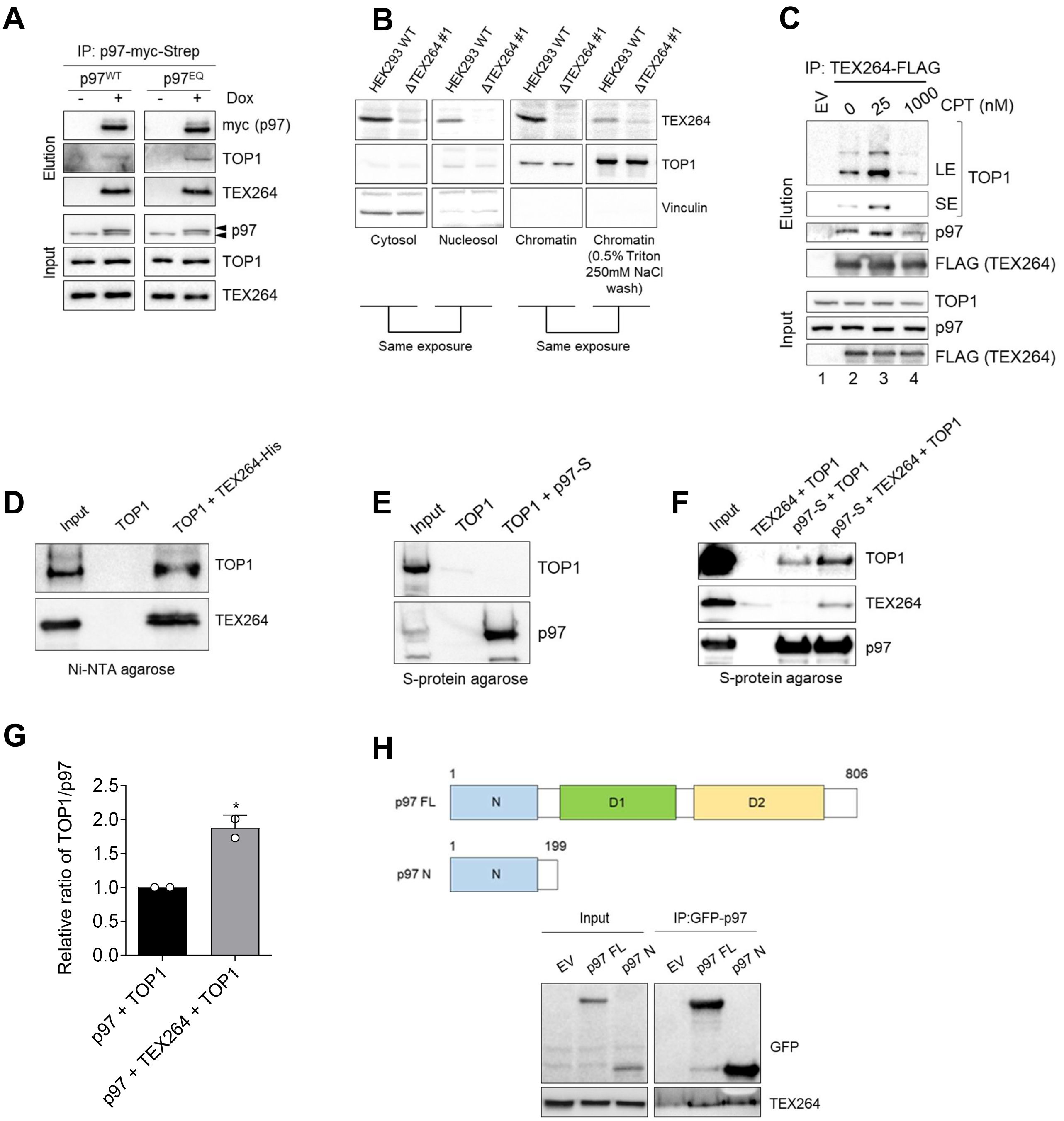

Supplementary Figure 2

Supplementary Figure 2: TEX264 Binds p97 and TOP1 *in vitro and in vivo*

A, Immunoblots of anti-Strep-tag immunoprecipitates prepared from doxycycline (Dox)-inducible HEK293 cells expressing p97^{WT-} or ^{EQ}-myc-Strep. Arrowheads indicate endogenous p97 (lower band) and p97-myc-Strep (upper band). **B,** Cytosolic, nuclear soluble (nucleosol), and chromatin fractions prepared from WT and TEX264-knockout (ΔTEX264) HEK293 cells immunoblotted with the indicated antibodies. Chromatin was washed with 0.5% Triton X-100 and 250 mM NaCl to separate loosely-bound and tightly-bound protein fractions. **C,** Analysis of TEX264-FLAG immunoprecipitates prepared from HEK293 cells treated with the indicated doses of CPT for 1 hour. LE, long exposure; SE, short exposure. **D,** *In vitro* interaction (pull-down) assay using recombinant TOP1 incubated with or without His-tagged TEX264 (amino acids 34-313). **E,** *In vitro* interaction assay using recombinant TOP1 incubated with or without S-tagged p97. **F,** Pull-down of recombinant p97-S after incubation with TOP1, with or without TEX264. **G,** Quantification of F (error bars represent mean ± SD; n = 2; *P < 0.05; Student's t-test). **H,** Above: schematic diagrams of p97 proteins used below. Below: immunoblots of anti-GFP immunoprecipitates prepared from HEK293 cells expressing the indicated GFP-tagged p97 constructs. Source data are available online.

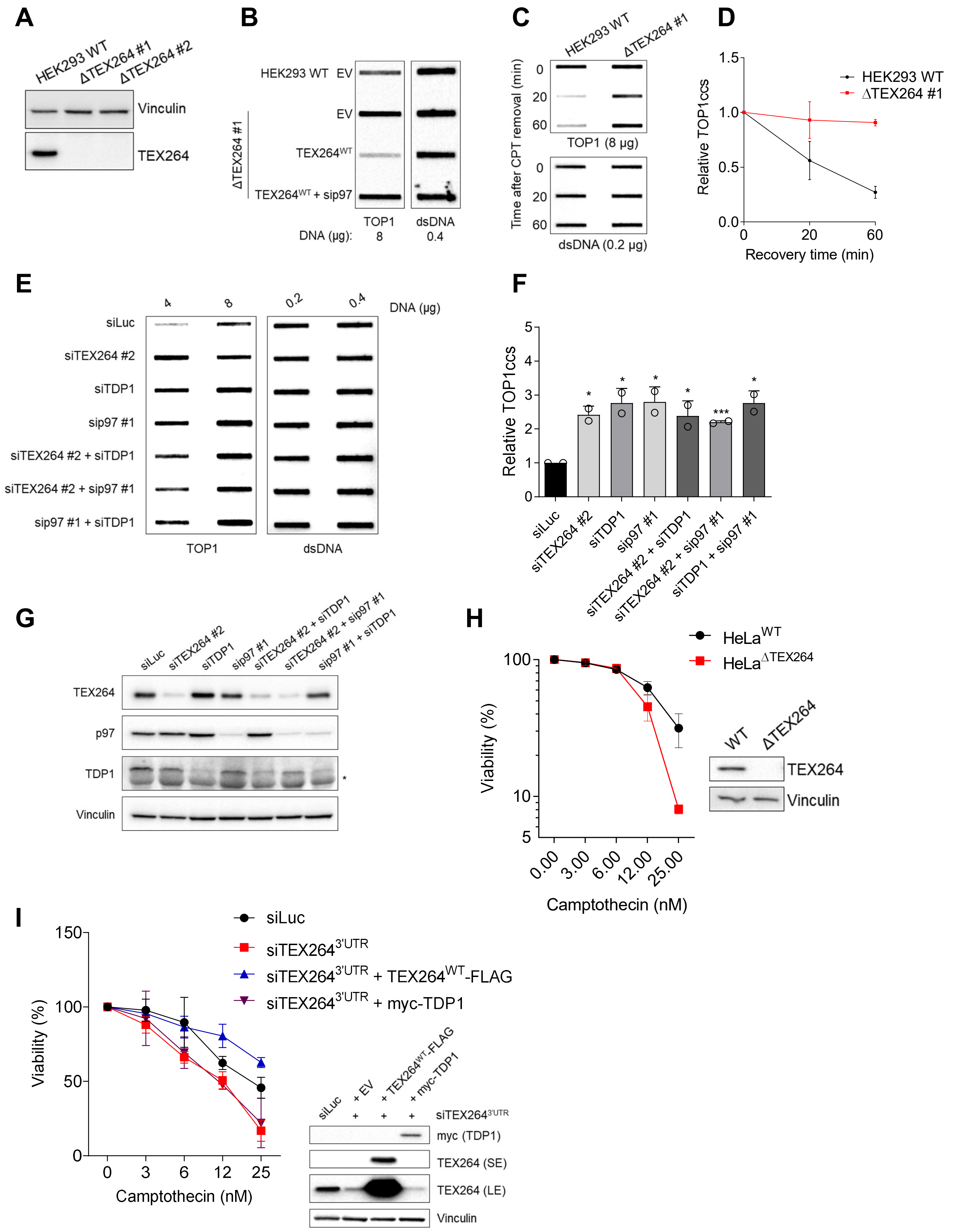

Supplementary Figure 3

Supplementary Figure 3: TEX264 Counteracts TOP1ccs

A, Immunoblot analysis of TEX264 expression in HEK293 WT and ΔTEX264 cells. **B,** RADAR analysis of TOP1ccs in WT and ΔTEX264 HEK293 cells treated with the indicated siRNAs and transfected with empty vector (EV) or cDNA encoding $TEX264^{WT}$-SSH. **C,** TOP1ccs isolated by RADAR from WT or ΔTEX264 HEK293 cells treated for 1 hour with CPT (50 nM), then released into CPT-free media for 20 or 60 minutes. **D,** Quantification of C (error bars represent mean± SEM; *n* = 3). **E,** RADAR analysis of TOP1ccs in HEK293 cells treated with the indicated siRNAs. **F,** Quantification of E (error bars represent mean ± SD; *n* = 2; **P* < 0.05; ****P* < 0.001; Student's t-test). **G,** Immunoblots to confirm depletions in E. The asterisk indicates an unspecific band. **H,** Colony forming assay to assess the survival of WT or ΔTEX264 HeLa cells after CPT treatment. Cells were treated for 24 hours with the indicated doses of CPT, then allowed to recover for 7 days (error bars represent mean ± SEM; *n* = 3). Viability is expressed as a percentage of the corresponding untreated samples. Right: corresponding immunoblots. **I,** Colony forming assay to assess the survival of HeLa cells after CPT treatment (performed as described for H). Right: control immunoblots (LE & SE denote long & short exposure, respectively). Source data are available online.

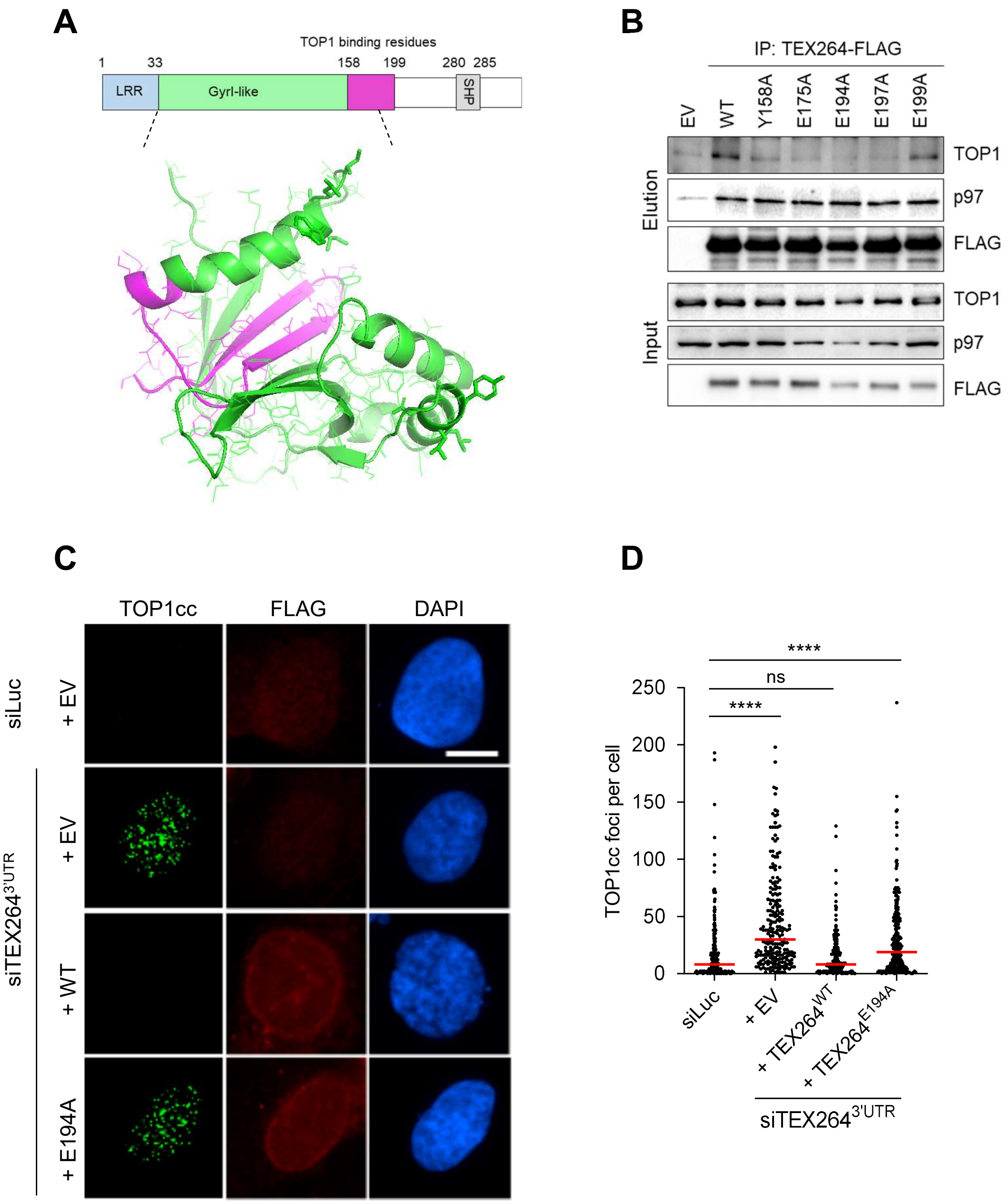

Supplementary Figure 4

Supplementary Figure 4: The GyrI-like Domain of TEX264 is Important for TOP1cc Repair

A, Above: schematic diagram of the TEX264 protein with the residues implicated in TOP1 binding indicated in pink. Below: structural model of the GyrI-like domain of TEX264 generated using Phyre2. Residues 158-184 are highlighted in pink. Residue 185-199 are outside of the model, as these residues are unique to TEX264. **B,** Immunoblots of FLAG immunoprecipitates prepared from ΔTEX264 HEK293 cells transiently expressing the indicated versions of FLAG-tagged TEX264. **C,** Representative images of U2OS cells treated with siLuc or siTEX264$^{3'UTR}$, transfected with the indicated FLAG-tagged variants of TEX264, and immunostained with TOP1cc (green) and FLAG (red) antibodies. Scale bar, 10 µm**. D,** Quantification of experiments represented in C. Red line indicates median. Data are representative of 3 independent experiments. Statistical analysis was performed using Kruskal-Wallis ANOVA with multiple comparisons, with Dunn's post-test. Source data are available online.

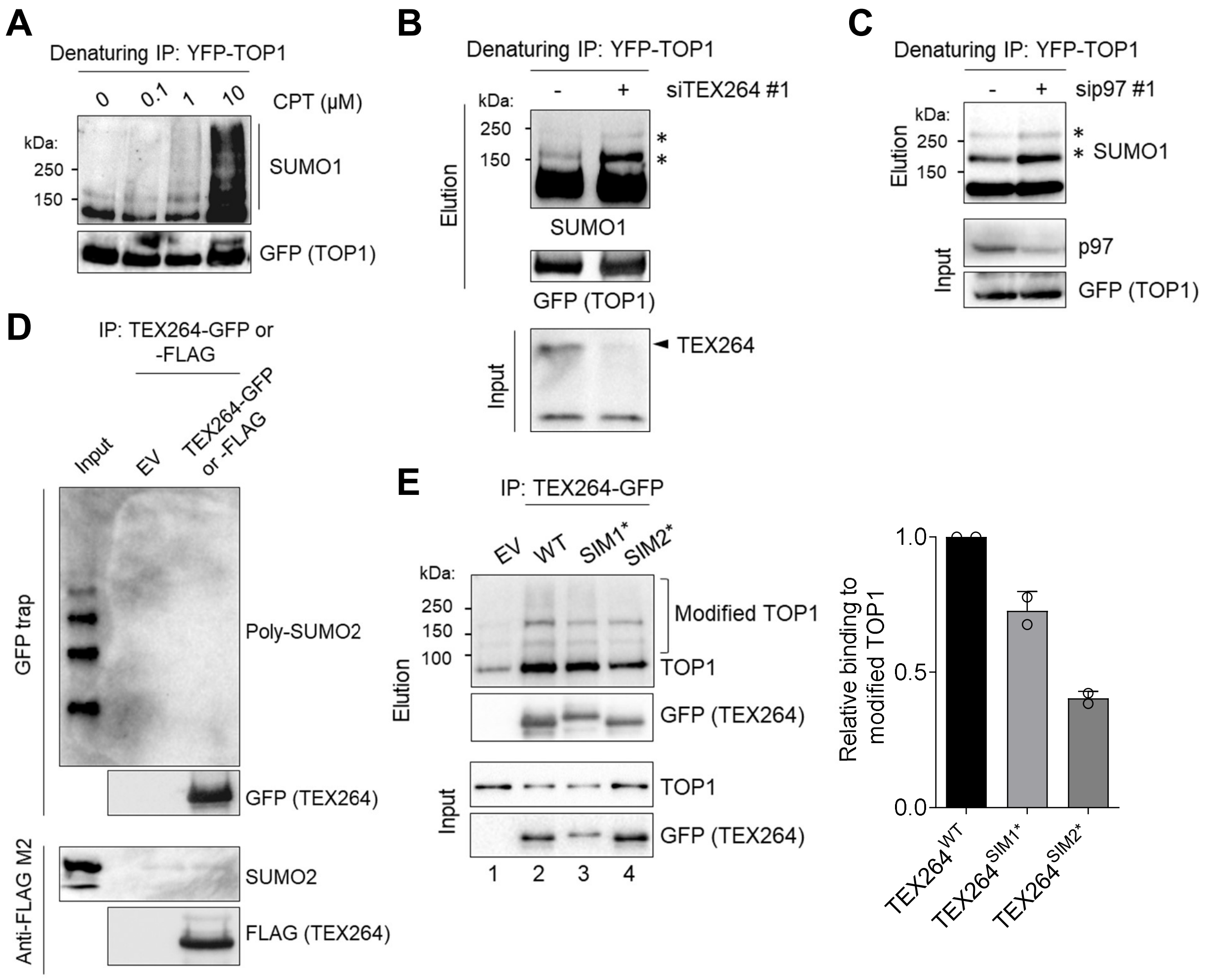

Supplementary Figure 5

Supplementary Figure 5: TEX264 Binds SUMO1-modified TOP1

A, Immunoblots of SUMO1-modified YFP-tagged TOP1. YFP-TOP1 was immunoprecipitated under denaturing conditions from HEK293 cells treated with the indicated doses of CPT for 1 hour. **B,** Immunoblot of SUMO1-modifed TOP1 after immunoprecipitation of YFP-TOP1 under denaturing conditions from HEK293 cells treated with the indicated siRNA. Asterisks indicate SUMO1-modified species of YFP-TOP1. **C,** Same as B. **D,** ΔTEX264 HEK293 cells transfected with EV or cDNA encoding either GFP- or FLAG-tagged TEX264 alleles were subjected to either anti-FLAG or -GFP IP under stringent conditions. Beads were incubated with either free, non-conjugated SUMO2 or poly-SUMO2 chains then immunoblotted with GFP, FLAG, or SUMO2/3 antibodies. **E,** Left panel: immunoblot analysis of GFP immunoprecipitates prepared from ΔTEX264 HEK293 cells transiently expressing the indicated GFP-tagged versions of TEX264. Right panel: corresponding quantification (mean ± SD; *n* = 2). Source data are available online.

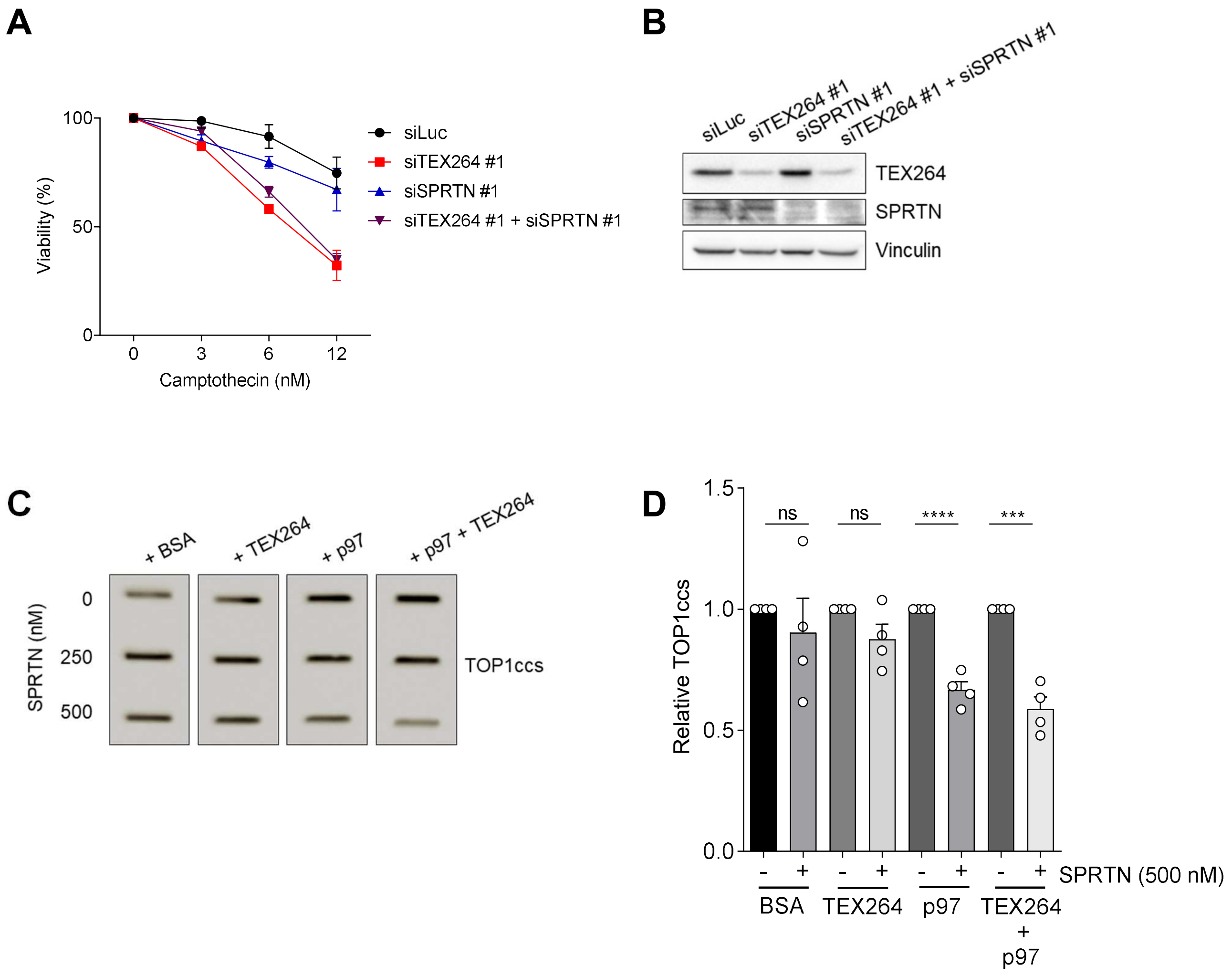

Supplementary Figure 6

Supplementary Figure 6: TEX264 Cooperates with the Metalloprotease SPRTN in TOP1cc Repair

A, Colony forming assay to assess the survival of HeLa cells transfected with the indicated siRNAs following a 24 hour treatment with the indicated doses of CPT (error bars represent mean ± SD; $n = 2$). **B,** Immunoblots to confirm efficacy of depletions in A. **C,** *In vitro* TOP1cc repair assay. Representative images of each condition are shown. TOP1ccs were purified from FlpIn T-REx HEK293 cells inducibly expressing TDP1-targetting miRNAs and treated with 10 μM MG132 for 1 hour and 14 μM CPT for 30 minutes. TOP1ccs were incubated with the indicated purified proteins, slot-blotted onto a nitrocellulose membrane and probed with a TOP1cc-specific antibody. **D,** Quantification of C (mean ± SEM; $n = 4$; ns, not significant; ***$P < 0.001$; ****$P < 0.0001$; Student's t-test). TOP1cc levels were normalised to the corresponding control sample (i.e. 0 nM SPRTN/reaction) in each condition. Source data are available online.

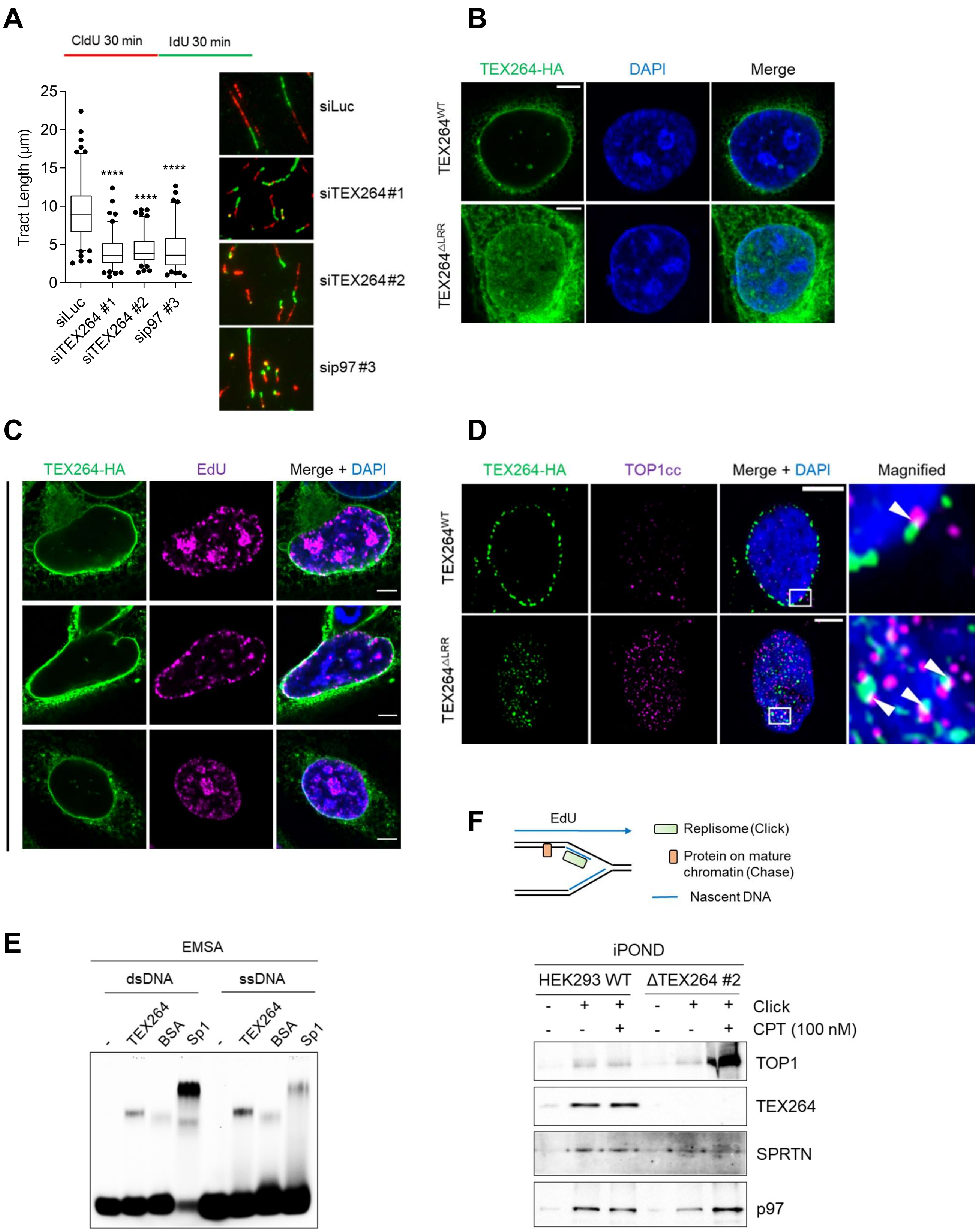

Supplementary Figure 7

Supplementary Figure 7: TEX264 Acts at Replication Forks

A, DNA fibre analysis of replication fork velocity in HEK293 cells treated with the indicated siRNAs. IdU track lengths are shown. Whisker box plots show median values and data within the 5–95 percentile. At least 100 fibres were measured per condition (****P < 0.0001; one-way ANOVA). Right: representative DNA fibres. **B,** Confocal images of U2OS cells transfected with the SSH-tagged TEX264 variants and stained with the indicated antibodies. Scale bar, 5 µm. **C,** Confocal images of U2OS cells transfected with cDNA encoding SSH-tagged TEX264 and stained with an anti-HA antibody. Cells were treated with 10 µM EdU for 30 minutes before fixation. EdU was labelled using a Click-iT™ Alexa Fluor imaging kit. Scale bars, 5 µm. See also Fig. 6C. **D,** Deconvolved confocal images (maximum intensity projections; 11 x 200 nm slices) of U2OS cells transfected with SSH-tagged TEX264 variants and stained with indicated antibodies. White arrowheads indicate examples of co-localisation. **E,** Electrophoretic mobility shift analysis (EMSA) to test for TEX264-His binding to single-stranded (ss) and double-stranded (ds) DNA. Consensus sequence DNA probes for the transcription factor, Sp1, were used. Storage buffer (-) and BSA were used as negative controls. Sp1 was used as a positive control. **F,** Above: schematic diagram of iPOND. Below: immunoblots of iPOND experiment performed in WT and ΔTEX264 HEK293 cells. Where indicated cells were treated with CPT (100 nM) for a total of 1 hour. Source data are available online.

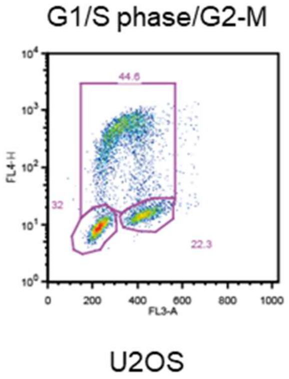

Supplementary Figure 8: Example of flow cytometry gating strategy for U2OS cells

FACS plot showing the gating strategy of U2OS in G1, S phase, and G2-M phase.

Supplementary Figure 8

Supplementary table 1 – Primers used in this study

Primer	Sequence	Purpose
TEX264_E175A_F	GCTGGAGATCTACCAGGCAGACCAGATCCATTTCA	Generation of TEX264 E175A variant
TEX264_E175A_R	TGAAATGGATCTGGTCTGCCTGGTAGATCTCCAGC	
TEX264_E194A_F	ACTCTGTCTCCTTCATCGCAGGCACATAGAAGTCT	Generation of TEX264 E194A variant
TEX264_E194A_R	AGACTTCTATGTGCCTGCGATGAAGGAGACAGAGT	
TEX264_E197A_F	CCATTTCCACTCTGTCGCCTTCATCTCAGGCAC	Generation of TEX264 E175A variant
TEX264_E197A_R	GTGCCTGAGATGAAGGCGACAGAGTGGAAATGG	
TEX264_E199A_F	CCCCGCCATTTCCACGCTGTCTCCTTCATCT	Generation of TEX264 E199A variant
TEX264_E199A_R	AGATGAAGGAGACAGCGTGGAAATGGCGGGG	
TEX264_Y158A_F	TCCTGCCTTGGACACCGCCATCAAGGAGCGGAAG	Generation of TEX264 Y158A variant
TEX264_Y158A_R	CTTCCGCTCCTTGATGGCGGTGTCCAAGGCAGGA	
TEX264_SIM1_round 1_F	CTTGAAGCCAAATTTCTGGGCGAGGGGCGATGGCCTCAGGGGAGGGCGATTC	Generation of TEX264 SIM1 variant
TEX264_SIM1_round 1_R	GAATCGCCCTCCCCTGAGGCCATCGCCCTCGCCCAGAAATTTGGCTTCAAG	
TEX264_SIM1_round 2 _F	CCAAATTTCTGGGCGGCGGCGGCGGCCTCAGGGGAGGG	
TEX264_SIM1_round 2 _R	CCCTCCCCTGAGGCCGCCGCCGCCGCCCAGAAATTTGG	
TEX264_SIM2_F	CAGGATGGACACGGCGGGTAGCCAGCGCGATGGCCAGAGCGGTGGTGTAGGGGAAGGTGGCTGTC	Generation of TEX264 SIM2 variant
TEX264_SIM2_R	GACAGCCACCTTCCCCTACACCACCGCTCTGGCCATCGCGCTGGCTACCCGCCGTGTCCATCCTG	
TEX264_SHPdeletion_F	CAGCGGCTCCTCTGAGTCACGGCTGG	Generation of TEX264 ΔSHP variant
TEX264_SHPdeletion_R	CCAGCCGTGACTCAGAGGAGCCGCTG	
TEX264_LRRdeletion_R	AATTGCGGCCGCCTCCTTGCCCTTCTCAGGGG	Generation of TEX264 ΔLRR variant
TEX264_LRRdeletion_F_pcDNA5	ATATGGATCCATGGGGTACTCAGGGCTACTGGCTG	Forward primer for pcDNA5
TEX264_LRRdeletion_F_pET21a	AATTCATATGTCGGACCTGCTACTACTGG	Forward primer for pET21a
p97_200STOP_F	CATCATAGCCTACTTAATTTAAGGACTCCTCCTCATCCTC	Generation of truncated p97 variant
p97_200STOP_R	GAGGATGAGGAGGAGTCCTTAAATTAAGTAGGCTATGATG	

Supplementary table 2 – siRNAs used in this study

siRNA	Sequence
siLuc	CGUACGCGGAAUACUUCGA
sip97 #1	AAGUAGGGUAUGAUGACAUUG
sip97 #2	AAGAUGGAUCUCAUUGACCUA
sip97 #3	AACAGCCAUUCUCAAACAGAA
siTEX264 #1	CTCATCGACCTCTACCAGAAA
siTEX264 #2	CGGCTGGAGATCTACCAGGAA
siTEX264 3'UTR	CUUCCAGGACCCAGAAUAA
siTOP1	GGUCCCUGUUGAGAAACGA
siTDP1	GGAUAUUGCGUUUGGAACA
siUBC9	GUGGCUGUCCCAACAAAAA
siSPRTN #1	AGCCAAUAUAACGGUAUACCA
siSPRTN 3'UTR	GUCAGGAAGUUCUGGUUAA

Supplementary table 3 – Antibodies used in this study

Antibody	Dilutions	Source	Identifier
HA [clone 3F10] (rat monoclonal)	1:5000	Roche	3F10; RRID:AB_2314622
PCNA (mouse monoclonal)	1:2000	Abcam	ab29; RRID:AB_303394
p97 (rabbit polyclonal)	1:1000	Proteintech	10736-1-AP; RRID:AB_2214635
p97 (rabbit polyclonal)	1:1000	In house	N/A
Topoisomerase 1 (rabbit polyclonal)	1:5000	Bethyl Laboratories	A302-589A; RRID:AB_2034865
Topoisomerase I-DNA Covalent Complexes Antibody, clone 1.1A (mouse monoclonal)	1:100	Millipore	MABE1084; ; RRID:AB_2756354
TEX264 (rabbit polyclonal)	1:1000	In house	N/A
TEX264 (mouse monoclonal)	1:1000	Novus	H00051368-M01; ; RRID:AB_2255844
Vinculin (mouse monoclonal)	1:2000	Bethyl Laboratories	ab18058; RRID:AB_444215
FLAG (rabbit polyclonal)	1:2000	Sigma-Aldrich	F7425; RRID:AB_439687
myc (mouse monoclonal)	1:1000	Cell Signaling Technology	2276; RRID:AB_331783
Double-stranded DNA (mouse monoclonal)	1:5000	Abcam	ab27156; RRID:AB_470907
SUMO1 (rabbit polyclonal)	1:1000	Abcam	ab11672; RRID:AB_298480
SUMO2/3 , clone 18H8 (rabbit monoclonal)	1:1000	Cell Signaling Technology	4971; RRID:AB_2198425
SPRTN N-terminal (rabbit polyclonal)	1:1000	In house; Lessel et.al, 2014	N/A
SPRTN C-terminal (rabbit polyclonal)	1:1000	Atlas	HPA025073
GFP	1:2000	Abcam	ab290; RRID:AB_303395
POLD1 (mouse monoclonal)	1:1000	Abcam	ab10362; RRID:AB_297099
MCM7 (141.2) (mouse monoclonal)	1:1000	Santa Cruz Biotechnology	sc-9966; RRID:AB_627235
TDP1 (rabbit polyclonal)	1:1000	Abcam	ab4166; RRID:AB_304337
Mono- and polyubiquitinylated conjugates, mAb (FK2)	1:1000	Enzo Life Sciences	BML-PW8810; RRID:AB_10541840
Tubulin (mouse monoclonal)	1:5000	Sigma-Aldrich	T6199; RRID:AB_477583
Histone H2A.X, phospho (Ser139; rabbit polyclonal)	1:200	Novus	NB 100-2280, RRID:AB_577933
Rabbit IgG, HRP-conjugated (goat polyclonal)	1:25,000	Sigma-Aldrich	A9169; RRID:AB_258434
Mouse IgG, HRP-conjugated (rabbit polyclonal)	1:25,000	Sigma-Aldrich	A9044; AB_258431
Mouse IgG, Alexa Fluor 488 (goat polyclonal)	1:500	Molecular Probes	A-11001; RRID:AB_2534069
Rabbit IgG, Alexa Fluor 594 (donkey polyclonal)	1:500	Thermo Fisher Scientific	R37119; RRID:AB_2556547
Mouse IgG, Alexa Fluor 488	1:500	Thermo Fisher Scientific	R37114; AB_2556542
Rat IgG, Cy3-AffinityPureF(ab')2 Fragment (donkey polyclonal)	1:300	Jackson ImmunoResearch Labs	712-166-153; RRID:AB_2340669
beta Actin (mouse monoclonal)	1:2000	Thermo Fisher Scientific	AM4302, RRID:AB_2536382
BrdU [clone BU1/75(ICR1)] (rat monoclonal)	1:500	Abcam	ab6326; RRID:AB_305426
BrdU [clone B44] (mouse monoclonal)	1:500	BD Biosciences	347580; RRID:AB_400326
