# Supplementary material for: TEX264 coordinates p97- and SPRTN-mediated resolution of topoisomerase 1-DNA adducts
Source: Nat Commun. 2020 Mar 9;11:1274. doi: 10.1038/s41467-020-15000-w (PMC7062751; doi:10.1038/s41467-020-15000-w)
Supplement: Supplementary file 2 — Reporting Summary [file 41467_2020_15000_MOESM2_ESM.pdf]

nature research

Corresponding author(s): NCOMMS-19-36542-T

Reporting Summary

Nature Research wishes to improve the reproducibility of the work that we publish. This form provides structure for consistency and transparency in reporting. For further information on Nature Research policies, see Authors & Referees and the Editorial Policy Checklist.

Statistical parameters

When statistical analyses are reported, confirm that the following items are present in the relevant location (e.g. figure legend, table legend, main text, or Methods section).

n/a	Confirmed	
☐	☒	The exact sample size (*n*) for each experimental group/condition, given as a discrete number and unit of measurement
☐	☒	An indication of whether measurements were taken from distinct samples or whether the same sample was measured repeatedly
☐	☒	The statistical test(s) used AND whether they are one- or two-sided<br>*Only common tests should be described solely by name; describe more complex techniques in the Methods section.*
☒	☐	A description of all covariates tested
☒	☐	A description of any assumptions or corrections, such as tests of normality and adjustment for multiple comparisons
☐	☒	A full description of the statistics including central tendency (e.g. means) or other basic estimates (e.g. regression coefficient) AND variation (e.g. standard deviation) or associated estimates of uncertainty (e.g. confidence intervals)
☒	☐	For null hypothesis testing, the test statistic (e.g. *F*, *t*, *r*) with confidence intervals, effect sizes, degrees of freedom and *P* value noted<br>*Give P values as exact values whenever suitable.*
☒	☐	For Bayesian analysis, information on the choice of priors and Markov chain Monte Carlo settings
☒	☐	For hierarchical and complex designs, identification of the appropriate level for tests and full reporting of outcomes
☒	☐	Estimates of effect sizes (e.g. Cohen's *d*, Pearson's *r*), indicating how they were calculated
☐	☒	Clearly defined error bars<br>*State explicitly what error bars represent (e.g. SD, SE, CI)*

Our web collection on statistics for biologists may be useful.

Software and code

Policy information about availability of computer code

Data collection	ImageLab (BioRad) v5.2.1<br>ImageJ v1.46r<br>Microscopy: Nikon 90i, Nikon Ti-E
Data analysis	GraphPad Prism v7<br>FlowJo<br>Andor Komet7.1<br>Andor Dragonfly 200

For manuscripts utilizing custom algorithms or software that are central to the research but not yet described in published literature, software must be made available to editors/reviewers upon request. We strongly encourage code deposition in a community repository (e.g. GitHub). See the Nature Research guidelines for submitting code & software for further information.

Data

Policy information about availability of data

All manuscripts must include a data availability statement. This statement should provide the following information, where applicable:

- Accession codes, unique identifiers, or web links for publicly available datasets
- A list of figures that have associated raw data
- A description of any restrictions on data availability

The mass spectrometry proteomics data have been deposited to the ProteomeXchange Consortium via the PRIDE partner repository with the dataset identifiers PXD017239 and 10.6019/PXD017239.

Field-specific reporting

Please select the best fit for your research. If you are not sure, read the appropriate sections before making your selection.

☒ Life sciences ☐ Behavioural & social sciences ☐ Ecological, evolutionary & environmental sciences

For a reference copy of the document with all sections, see nature.com/authors/policies/ReportingSummary-flat.pdf

Life sciences study design

All studies must disclose on these points even when the disclosure is negative.

Sample size	Most experiments were reproduced at least three times with similar results. For DNA fibre analysis, at least 100 fibres per sample were measured. For foci number & nuclear intensity studies, at least 100 cells were analysed.
Data exclusions	No relevant data was excluded from this study.
Replication	Experiments were performed multiple times. The representative experiments presented in the manuscript were typically performed in 2-3 biological replicates. For most experiments (e.g. DNA fibre analysis, comet assay, colony forming assays and immunofluorescence) each biological replicate included 2-3 technical replicates.
Randomization	In each experiment, different cell samples started from similar conditions and treatments were randomly allocated.
Blinding	Biological replicate for experiments in Fig. 3A-B were carried out blindly.

Reporting for specific materials, systems and methods

Materials & experimental systems

n/a	Involved in the study	
☒	☐	Unique biological materials
☐	☒	Antibodies
☐	☒	Eukaryotic cell lines
☒	☐	Palaeontology
☒	☐	Animals and other organisms
☒	☐	Human research participants

Methods

n/a	Involved in the study	
☒	☐	ChIP-seq
☐	☒	Flow cytometry
☒	☐	MRI-based neuroimaging

Antibodies

Antibodies used	A full description of all antibodies used in this study is provided in Supplementary Information.
Validation	In this study, we have validated homemade anti-p97 and anti-TEX264 antibodies. The specificity of each antibody was confirmed by depleting the corresponding target protein using siRNA.<br>For all other antibodies, validation was performed by the manufacturers.

Eukaryotic cell lines

Policy information about cell lines

Cell line source(s)	All human cell lines originally from ATCC. Flp-In T-REx 293 cells were purchased from Thermo Fisher Scientific.
Authentication	U2OS, HeLa, HEK293, Flp-In T-REx HEK293, and RPE1 cells used in this study were originally obtained from ATCC and not further genetically authenticated.
Mycoplasma contamination	All cell lines used in this study were tested negative to mycoplasma by the MycoAlertTM Mycoplasma Detection Kit (Lonza LT07-218).
Commonly misidentified lines (See ICLAC register)	No misidentified cell lines were used in this study.

Flow Cytometry

Plots

Confirm that:

☒ The axis labels state the marker and fluorochrome used (e.g. CD4-FITC).

☒ The axis scales are clearly visible. Include numbers along axes only for bottom left plot of group (a 'group' is an analysis of identical markers).

☒ All plots are contour plots with outliers or pseudocolor plots.

☒ A numerical value for number of cells or percentage (with statistics) is provided.

Methodology

Sample preparation	For cell cycle analysis, TEX264 was depleted by siRNA for 72 hours. Cells were incubated with 10 µM EdU for 30 minutes before collection by trypsinisation. Cells were washed with PBS cells were incubated with EdU (Thermo Fisher) at a final concentration of 10 µM for 30 minutes. Cells were collected, washed twice in 1x PBS, resuspended in 4% PFA, and incubated for 15 minutes. Cells were then washed in PBS, resuspended in FACS buffer (0.25% Saponin, 1% FBS in 1x PBS) by vortexing, and incubated for 15 minutes. The Click reaction carried out according to the manufacturer's instructions using the Click-iT® EdU Alexa Fluor® 647 imaging kit (Thermo Fisher). Cells were washed once in FACS buffer and resuspended in 1% BSA (diluted in PBS) containing 10 µg/µL RNAse and 20 µg/mL Propidium Iodide and incubated for 30 minutes.
Instrument	FACScalibur (BD Biosciences)
Software	Data was analyzed with FlowJo.
Cell population abundance	The percentage of cells in each cell cycle phase was assessed and plotted
Gating strategy	Only isolated alive cells were analyzed.

☒ Tick this box to confirm that a figure exemplifying the gating strategy is provided in the Supplementary Information.
